# Immature monocytic cells within tumors differentiate into immunosuppressive cells in resistant tumors to immunotherapy

**DOI:** 10.1016/j.isci.2025.113141

**Published:** 2025-07-17

**Authors:** Sapir Levin, Madeleine Benguigui, Bar Manobla, Chen Buxbaum, Ziv Raviv, Keren Yizhak, Yuval Shaked

**Affiliations:** 1 Rappaport Faculty of Medicine, Technion – Israel Institute of Technology, Haifa, Israel

**Keywords:** immunology, cell biology, cancer

## Abstract

Immune checkpoint inhibitors (ICIs) have improved outcomes in advanced cancers, yet resistance remains a major obstacle. Here, we investigated the role of myeloid cells in shaping the immunosuppressive tumor microenvironment that contributes to ICI resistance. Using mutagenized ICI-sensitive and resistant 4T1 breast cancer clones, we performed single-cell RNA sequencing to characterize immune cell populations post-ICI therapy. We identified monocytic dendritic progenitors (MDPs) and common monocytic progenitors (cMOPs) enriched in sensitive tumors, which may differentiate into immunosuppressive cells in resistant tumors. Analysis of public datasets confirmed the presence of MDP-cMOPs in tumors and blood of patients with breast, lung, and colorectal cancer. We found high expression of CXCR4 and IL6R in MDP-cMOPs, and inhibiting these pathways blocked their recruitment and differentiation. Combined targeting of CXCR4 and IL6 pathway with ICI improved responses in resistant tumors, highlighting MDP-cMOPs as contributors to immunotherapy resistance and potential therapeutic targets.

## Highlights

• MDP-cMOPs accumulate in ICI-sensitive tumors
• In resistant tumors, MDP-cMOPs differentiate into immunosuppressive macrophages
• Blocking CXCR4 and IL-6 impairs MDP-cMOP recruitment and differentiation to tumors
• Inhibiting MDP-cMOPs improves ICI therapy efficacy in a resistant breast cancer model

## Graphical abstract

**Figure:**
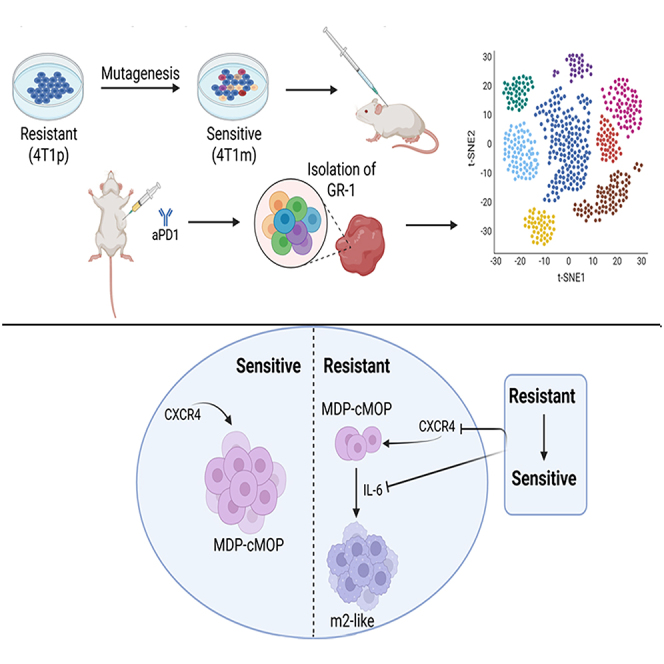

## Introduction

The field of immune checkpoint inhibitors (ICIs) has witnessed remarkable advancements over the past decade, significantly prolonging the response duration in patients with advanced metastatic disease.^1^ However, a significant hurdle that remains is the lack of response to this therapy in a substantial proportion of patients, mostly due to inherent resistance mechanisms.^1^ Furthermore, some patients witness an initial response followed by a rapid escape from therapy.^2^ Multiple mechanisms have been proposed to elucidate resistance to immunotherapy or relapse, including the lack of tumor antigen presentation, activation of bypass immune checkpoints, genetic and epigenetic alterations, and modifications in the tumor microenvironment, such as the recruitment of immunosuppressive cells or impaired cytotoxic T cell function.^2^ The latter extrinsic mechanism involves the infiltration of not only anti-tumor immune cells but also pro-tumorigenic immunosuppressive cells that contribute to increased tumor aggressiveness following ICI therapy.^3^^,^^4^ Key contributors to ICI resistance include myeloid-derived suppressor cells (MDSCs) and tumor-associated macrophages (TAMs), which can comprise up to 50% of the immune cell population in patients with breast cancer.^5^^,^^6^ However, the precise mechanisms by which tumors promote immunosuppressive cell colonization further contributing to ICI resistance remain unclear.

The therapeutic efficacy of ICIs depends on the interactions between tumor and host cells within the tumor microenvironment.^7^ Several studies demonstrated that predictive biomarkers for ICI therapy are associated with cellular states or gene expression patterns.^8^ In this regard, we previously identified a newly defined subset of Ly6E-high neutrophils that can predict response to ICI therapy.^9^ Specifically, these neutrophils are interferon-stimulated cells and release pro-inflammatory factors that enhance anti-tumor immunity and support favorable treatment outcomes.^9^ We also demonstrated that in ICI resistant tumors, granulocytic cells fail to progress beyond the immature state to the terminal state of Ly6E neutrophils. These findings suggest that the maturation state of immune cells within tumors may be associated with ICI responsiveness.

The immature myeloid cells (IMCs), which include myeloid progenitors and precursors of myeloid cells, are part of the normal process of myelopoiesis. This process is regulated by growth factors such as GM-CSF, G-CSF, and M-CSF, along with pro-inflammatory cytokines, particularly during inflammatory processes.^10^ Within the myeloid lineage, common myeloid progenitors (CMPs) further differentiate into granulocytic monocytic progenitors (GMPs) and subsequently to monocytic dendritic progenitors (MDPs), although the latter can independently differentiate from CMPs.^11^ GMPs and MDPs differentiate into functionally distinct monocytes: monocytic progenitors (MPs) and common monocytic progenitors (cMOP), respectively.^11^ In cancer or chronic inflammation, the myelopoiesis process becomes disrupted due to the persistent expression of pro-inflammatory cytokines. Tumor microenvironmental factors such as IL-1β, IL-6, TNF-α, and IFN-γ, promote IMCs accumulation, inhibit their differentiation, and activate their immunosuppressive functions, at which point they are referred to as MDSCs.^5^^,^^12^

There are various mechanisms by which MDSCs suppress the immune response. They can directly inhibit T cell cytotoxicity by upregulating PD-L1 or, indirectly, by inducing regulatory T cells.^5^ However, they are not the only cells within the tumor microenvironment that support tumorigenesis. Recent studies demonstrated that IMCs, and in particular MDPs, can differentiate into immunosuppressive macrophages that, in turn, secrete anti-inflammatory factors such as IL-10 and TGF-β, further supporting tumor growth and metastasis.^13^^,^^14^ We previously found that IL-6 signaling promotes the differentiation of MDPs into immunosuppressive macrophages rather than dendritic cells, further explaining their pro-metastatic role in cancer.^13^ We also found that blocking IL-6 not only reduced cancer metastasis but also improved the overall effectiveness of ICI therapy.^3^ Despite these aforementioned studies, little is known about the mechanisms by which tumors promote the recruitment and differentiation of immunosuppressive myeloid cells to support tumor growth and immune evasion. In this study, we investigate the role of IMCs in the tumor microenvironment of mice sensitive or resistant to immunotherapy, and their differentiation pattern which may explain immunosuppressive tumor microenvironment.

## Results

### Characterization of GR-1 myeloid cells in tumors sensitive and resistant to anti-PD1 therapy

To investigate tumors sensitive and resistant to immunotherapy, we used an experimental model of the 4T1 cell line, a triple negative breast carcinoma known for its resistance to ICI therapy.^9^ Previous studies have shown that increasing the mutational burden in cancer cells can enhance their response to immunotherapy.^9^^,^^15^ Following this, we exposed 4T1 cells to 1-methyl-3-nitro-1-nitrosoguanidine (MNNG) and generated polyclonal cells that would respond to anti-PD1 (referred to as 4T1m) as opposed to the resistant (parental) clone (referred to as 4T1p). The cells were then implanted into the mammary fat pad of mice and subsequently treated with anti-PD1 or IgG control. Similar to a previous study using this approach,^9^ mice bearing 4T1m tumors displayed a significant reduction in tumor growth following anti-PD1 therapy as opposed to mice bearing 4T1p tumors, indicating that 4T1m tumors were sensitive while 4T1p tumors were resistant to immunotherapy (Figure S1). At the endpoint (following two weeks of treatment), both 4T1p and 4T1m tumor-bearing mice treated with anti-PD1 were harvested and processed into single-cell suspensions for further analysis, as outlined below.

Persistent expression of inflammatory factors produced within the tumor microenvironment, including GM-CSF, G-CSF, IL-6, IL-1β, TNF-α, and IFN-γ, is known to promote the expansion of immunosuppressive cells.^12^ These same inflammatory factors also typically support the normal differentiation and migration of IMCs, even in the context of cancer.^16^^,^^17^^,^^18^ Performing cytokine array comparing 4T1m and 4T1p, we observed significantly higher expression levels of inflammatory cytokines, including GM-CSF, TNF-α, and IL-6, in 4T1m compared to 4T1p tumors (Figure S2). We hypothesized that the elevated cytokine levels in sensitive tumors could stimulate the migration of IMCs to the tumor microenvironment, which may subsequently differentiate into cells with either pro-tumoral or anti-tumoral functions.

**Figure 1:**
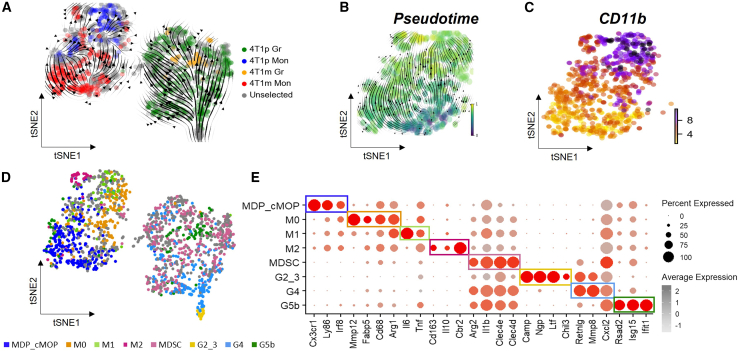
Characterization of GR1+ cells in 4T1m and 4T1p tumors following anti-PD1 therapy 4T1m (immunotherapy-responsive) and 4T1p (immunotherapy-resistant) tumors were harvested two weeks after treatment with anti-PD1 and were prepared as single cell suspensions. Subsequently, GR-1+ cells were isolated using magnetic beads, and processed for transcriptome analysis at a single cell level, using the 10× Genomic platform. (A) tSNE plot of 1959 GR-1+ cells, with 910 cells are monocytes (left cluster) and 1049 cells are granulocytes (right cluster). Cells are colored according to their classification as differentially abundant in the groups: 4T1p Gr and 4T1m Gr (granulocytes), 4T1p Mon and 4T1m Mon (monocytes), and unselected cells. (B) RNA velocity and pseudotime trajectory of the monocytic population, illustrating the maturation pattern with directional arrows indicating cell state progression. (C) mRNA expression of Itgam (CD11B), as determined by RNA velocity in the monocytic population, demonstrated higher expression in the mature monocytic state. (D) tSNE plot of cells annotated by the identified cell types. (E) A dotplot summarizing the expression of key marker genes across identified cell types, validating cell classification via a semi-supervised approach.

Typically, GR-1^+^CD11B^+^ cells are analyzed to study MDSCs^19^; however, recent studies have identified the expression of Ly6C on other IMCs, including granulocytic monocytic progenitors (GMPs) and monocytic dendritic progenitors (MDPs).^20^^,^^21^ Moreover, GMPs were shown to give rise to LKS^−^Ly6C^+^CD115^hi^ progenitors, indicating that while GR-1 is included among lineage-negative (Lin^−^) cells, Ly6C itself does not necessarily denote lineage-negative identity.^22^ This further suggests that GR-1+ cells can include both non-active IMCs (CD11B^−^), and immunosuppressive IMCs, referred to as MDSCs. Therefore, we isolated cells from anti-PD1-treated 4T1p and 4T1m tumors based on the GR-1 marker, which collects both Ly6C and Ly6G surface markers in mice.^23^ Data visualization revealed two distinct GR-1+ cell populations in tumor samples, corresponding to monocytic and granulocytic clusters in both 4T1p and 4T1m tumors^24^ (Figure 1A).

In this study, we first leveraged the association of GR-1 with myeloid cell differentiation to examine the diverse subsets within the population and their conditions, including their maturation stage. We aimed to analyze the dynamic accumulation and differentiation patterns of myeloid cells across each cluster. To accomplish this, we implemented two computational tools: (a) RNA velocity analysis, which recovers directional information by distinguishing newly pre-mRNA from mature mRNA.^25^ Projected arrows in the tSNE plot illustrate maturation trajectories for monocytic (left) and granulocytic (right) cells (Figure 1A). (b) Differential abundance (DA) analysis, performed with the DAseq package,^26^ was used to test unbiasedly which cells are depleted or enriched under the different conditions. Four distinct regions were identified - two for each condition (i.e., 4T1m and 4T1p tumors) - corresponding to both monocytic and granulocytic cells (Figure 1A). The “Unselected” cells refer to cells that were not specifically enriched in any particular condition. Our previous study on naive 4T1p and 4T1m samples revealed that immunotherapy resistance manifests at an earlier developmental stage within the granulocytic lineage, inhibiting the differentiation into Ly6E neutrophils and thereby supporting immunotherapy resistance.^9^ Consistently, the cells from the sensitive tumors to anti-PD1 (4T1m) are indeed located at the terminal stage of the granulocytic lineage (Figures 1A and S3A). Using downstream analyses of DA, we identified differentially expressed genes (DEGs) in granulocytic cells and found that DA-enriched cells in 4T1m tumors expressed high levels of interferon-inducible genes and *Ly6E*, consistent with our previous findings^9^ (Figure S3B; Table S1). In contrast, our updated analysis of anti-PD1-treated monocytic cells revealed a significant proportion of cells from 4T1m tumors occupying a pre-mature monocytic cluster (Figures 1A and 1B). Notably, these cells lacked *CD11b* expression, suggesting they represent non-activated IMCs rather than MDSCs (Figure 1C). Unsupervised clustering revealed that this pre-mature population was distributed across two subclusters (5 and 0), both expressing genes associated with dendritic cells or macrophages, but lacking granulocyte-associated markers (Figure S4). These included *C1q* family members and MHC class II genes such as *H2-Ab1* and *H2-Eb1*, which are characteristic of macrophages and dendritic cells^27^ (Figure S4). Semi-supervised clustering using known marker genes identified immune populations including various macrophage subsets (M0, M1, M2) and neutrophil populations at different stages of maturation (G2–G5b), consistent with previous classifications^28^ (Figure S5). Based on our analysis, we identified a cluster representing monocytic dendritic progenitors (MDPs) and common monocyte progenitors (cMOPs), as these cells express dendritic cell- or macrophage-associated genes, but not granulocyte markers (Figures S4 and S5). This cluster also lacked expression of genes associated with terminal maturation, such as *CD11b* (Figure 1C). The presence of *Cx3cr1*, *Ly86*, and *Irf8* in this cluster further supports its identity as an MDP–cMOP population^22^ (Figures 1D and 1E). Pathway enrichment analysis indicated that the MDP–cMOP cluster exhibits stem/progenitor-like features, consistent with their immature phenotype (Figure S6 and Table S2). Finally, DEGs analysis comparing MDP–cMOPs with other IMCs—including MDSCs (Figure S7A and Table S3) and M0 macrophages (Figure S7B and Table S4)—highlighted signature genes such as *Cx3cr1* and *Ly86*, consistent with previous reports.^11^^,^^22^ Taken together, our single-cell analysis suggests that MDP–cMOPs are present in ICI-treated tumors.

### Monocytic-dendritic progenitors and common monocytic progenitors tend to accumulate in sensitive tumors

To confirm that MDP-cMOP cells are present in tumors, we characterized cell types from healthy bone marrow (Figure S8A) and integrated them with the tumor MDP-cMOP cluster (Figure S8B; see STAR Methods) to distinguish them from MDSCs. Indeed, we found that the MDP-cMOP cluster is closely related to MDPs derived from healthy bone marrow, further suggesting that these were true MDP-cMOPs (Figure S8B). Interestingly, while MDPs in the bone marrow exhibited gene signatures associated with hematopoietic stem and progenitor cell (HSPC) differentiation, such as Hmgb3 and Plac8,^29^^,^^30^ in the tumors, they displayed genes associated with their differentiation into M2-like macrophages, e.g., Gatm and Mafb^31^^,^^32^ (Figure S8C).

**Figure 2:**
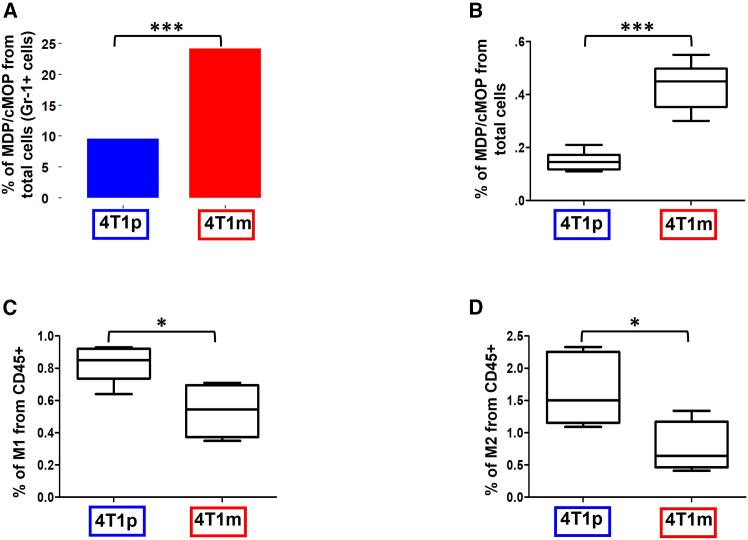
Enrichment of MDP-cMOPs in responsive tumors (A) The frequency of MDP-cMOPs was quantified as a percentage of total GR-1+ cells between 4T1p and 4T1m tumors. Statistical comparisons were performed using the BCa bootstrap independent two-samples test for proportion differences. (B–D) 4T1p and 4T1m tumor-bearing mice were treated with anti-PD1. After 2 weeks, tumors were removed and prepared as single-cell suspensions. The percentage of MDP-cMOPs (B), M1-like (C), and M2-like (D) macrophages was analyzed by flow cytometry. The gating strategies are shown in Figures S1 and S2. Data are presented as mean ± standard deviation (SD). Statistical significance was assessed by an unpaired two-tailed t-test. Significant *p*-values are shown as ∗*p* < 0.05, ∗∗∗*p* < 0.001.

To validate these findings, we analyzed tumor samples from mice bearing 4T1p or 4T1m tumors treated with anti-PD1. Given the rarity of these cells, we utilized flow cytometry to detect MDP-cMOPs without separating MDPs from cMOPs, aligning with our scRNA-seq analysis. While cMOPs express Ly6C and MDPs express CD135,^11^ our flow cytometry approach identified both populations together using the surface marker combination SCA1^-^CD117^+^CD34^+^CD115^+^ (Table S5). The single-cell analysis demonstrated that the abundance of MDP-cMOPs between tumors resistant and sensitive to anti-PD1 was over 2-fold higher in responding tumors (Figure 2A). These results were also confirmed in mice using flow cytometry, showing that MDP-cMOP frequency was higher in the 4T1m tumors compared to the 4T1p tumors (Figures 2B and S9 for gating strategy). In contrast, the analysis of subsets of macrophages revealed that M1-and M2-like macrophages were more abundant in the resistant tumors compared to the more sensitive ones (Figures 2C, 2D, and S10 for gating strategy). We, therefore, hypothesized that MDP-cMOPs might accumulate in sensitive tumors, where they have no immunomodulatory function. However, in tumors resistant to ICI, these cells may potentially differentiate into immunosuppressive monocytic cells (e.g., MDSCs and M2 macrophages), further explaining therapy resistance.

### Monocytic-dendritic progenitors and common monocytic progenitors are pre-committed to immunosuppressive monocytic cells

**Figure 3:**
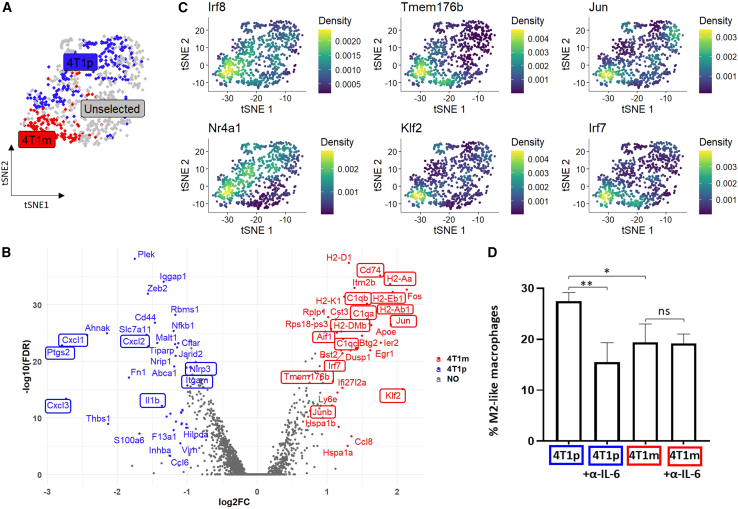
Differential abundance and gene expression of monocytic cells in 4T1m versus 4T1p tumors (A) tSNE plot of 910 filtered, GR-1+ monocytic cells, with cells colored based on differential abundance score between 4T1m (red) and 4T1p (blue). (B) DEGs between 4T1m and 4T1p (also shown in Table S4) are presented in a volcano plot (FDR< 0.01, and log_2_ fold-change >1 or log_2_ fold-change < −1). Notably, genes associated with MDP and cMOP cluster are highlighted. (C) Binned, normalized expressions of MDP-cMOP marker genes are shown. (D) The percentage of M2-like macrophages analyzed after MDP-cMOPs were cultured in the presence of conditioned medium from 4T1p or 4T1m tumors with or without anti-IL-6 antibodies. Data are presented as mean ± standard deviation (SD). Statistical significance was assessed using one way ANOVA followed by Tukey post-test. Only comparisons of interest are shown. ns, non-significant. Significant *p* values are shown as ∗*p* < 0.05 and ∗∗*p* < 0.01.

To further investigate the differences between 4T1p and 4T1m tumors in relation to monocytic cells, we performed differential expression analysis between the two DA regions (4T1m-Mon vs. 4T1p-Mon, from Figure 1A). We found higher expression of *CD11b* (*Itgam*) in the 4T1p-Mon cluster compared with the 4T1m-Mon cluster (Figures 3A and 3B; Table S6). This finding indicates that 4T1p tumors contain a greater proportion of more mature monocytic cells compared to 4T1m tumors. We also observed elevated expression of genes associated with inflammatory monocytes, such as Il1b and Nlrp3,^33^ as well as chemokines such as Cxcl1, Cxcl2, and Cxcl3.^34^ Additionally, *Ptgs2*, a gene representing a factor known to promote the generation of immunosuppressive macrophages from monocytes,^35^ was highly expressed in this cluster, consistent with the maintenance of an immunosuppressive microenvironment, as expected in resistant tumors (Figure 3B). Importantly, when analyzing cells enriched in 4T1m tumors, we found the same genes associated with the MDP–cMOP population—such as *C1q* and MHC class II genes—and also identified genes linked to their differentiation into M2-like macrophages, including *Irf7*, *Jun*, and *Klf2*^36^^,^^37^ (Figures 3B and 3C). Furthermore, TMEM176b, known to inhibit inflammasome and suggested to be a negative regulator of dendritic cell maturation,^38^ was overexpressed in this cluster. This further indicates the commitment of MDP-cMOPs to immunosuppressive M2-like cells rather than to dendritic cells (Figures 3B and 3C). Notably, we found that both sensitive and resistant tumors co-express Irf8 and Nr4a1, which are involved in the differentiation of MDP-cMOPs into non-classical M2 monocytes (Figure 3C).^11^ To further support our computational analysis, we isolated MDP-cMOPs from the bone marrow of naive mice, and cultured them with 4T1p or 4T1m conditioned medium (CM). As IL-6 has been implicated in promoting the differentiation of MDPs into immunosuppressive macrophages,^13^ we also evaluated the impact of IL-6 inhibition in this setting. After two days, cells were analyzed by flow cytometry for M2 macrophage surface markers. We observed that culturing with 4T1p CM led to a higher percentage of M2-like macrophages compared to 4T1m CM. The addition of anti-IL-6 antibodies reduced the number of M2-like macrophages only in the 4T1p CM, further indicating that MDP-cMOPs can differentiate into immunosuppressive macrophages in resistant tumors (Figure 3D). Overall, these data suggest that MDP-cMOPs are pre-committed to M2-like macrophages in the presence of factors secreted by resistant tumors.

### Identification of monocytic-dendritic progenitors and common monocytic progenitors in tumors of patients with cancer

**Figure 4:**
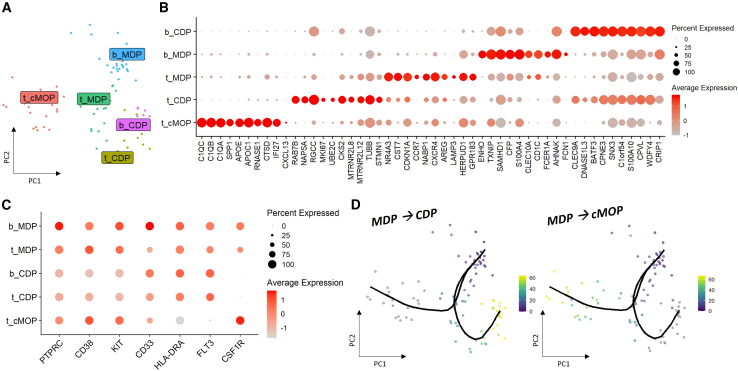
MDPs in patients with breast cancer treated with immunotherapy (A) A PCA plot of 94 cells represent CD38 and CD117 positive cells is shown. Cells originating from tumor (t) or blood (b) are colored by cell type. (B) A dotplot demonstrates the top 10 differentially expressed genes for each cluster. (C) The surface marker gene expression of MDPs (CD45RA^+^CD38^+^CD117^+^HLA-DRA^+^FLT3^+^CSF1R^+^), CDPs (CD45RA^+^CD38^+^CD117^+^HLA-DRA^+^FLT3^+^CSF1R^−^), and cMOPs (CD45RA^+^CD38^+^CD117^+^HLA-DRA^+^FLT3^−^CSF1R^+^) are indicated. (D) Trajectory analysis reveals the two lineages of MDPs and their committed cell progeny: CDP (left) and cMOP (right).

While MDP-cMOPs were identified and validated in mice, a question remains regarding their existence in tumors of patients with cancer, further testifying to their clinical role. Our previous study identified MDPs in the blood of patients with breast and lung cancers using markers indicated in Table S5.^13^ We therefore sought to analyze MDPs in human specimens. Specifically, we identified two datasets of cells from patients with breast cancer who underwent immunotherapy, chemotherapy, or a combination thereof. These datasets were merged for the analysis of MDPs at the tumor and/or peripheral blood (see STAR Methods, and Figure S11 for a lack of batch effect). We assessed the expression of CD117 and CD38 markers, known markers of hematopoietic progenitor cells,^39^ mast cells,^40^ and lymphocytes,^41^ but not other mature myeloid cells. Using these markers, we identified a subpopulation of MDP-derived cells in both the blood and tumors of patients with breast cancer (Figure 4A). Unsupervised clustering of CD117^+^CD38^+^ myeloid cells revealed subgroups expressing genes representing surface markers consistent with MDPs, common dendritic progenitors (CDPs), and cMOPs, including CD45R, CD33, HLA-DRA, FLT3, and CSF1R (Figures 4B and 4C). To investigate the differentiation potential of these progenitor populations, we performed trajectory analysis using the *Slingshot* package.^42^ The analysis indicated that MDPs from both blood and tumors may give rise to either CDPs or cMOPs (Figure 4D). This contrasts with the murine data, in which tumor-resident MDPs appeared committed exclusively to immunosuppressive monocytic lineage. We hypothesize that this difference may be attributed to the lack of GR-1 or other potential common gene expression in CDPs, which complicates their detection in mouse datasets. Supporting this, gene expression dynamics along the trajectory from MDPs to cMOPs showed a continuous transition (Figure S12A), whereas a pronounced gap was observed between MDPs and CDPs (Figure S12B). These findings suggest that, in tumors, MDPs are more likely to give rise to cMOPs, while CDPs may originate from circulating blood rather than from tumor-resident progenitors. Taken together, this analysis supports the notion that cMOPs are predominantly derived from tumor-localized MDPs, whereas CDPs found in tumors may reflect infiltration from peripheral blood.

Next, comparing the human and mouse data, we found similar genes expressed by MDPs committed to cMOPs, including members of the C1q family and Aif1 (Figure S13A), as well as dendritic cell-related genes such as HLA, CD74, and CST3, expressed by MDPs committed to CDPs (Figure S13B). Notably, additional genes including IRF7 and TMEM176B were also identified in human MDP and cMOP populations, consistent with the murine data (Figure S13C). MDPs and cMOPs were also observed in additional colorectal^43^ and lung cancer^44^ patient datasets (Figure S14), further supporting a pan-cancer activity of MDP–cMOPs. These aforementioned genes in colorectal cancer (Figure S15), and lung cancer (Figure S16), highlight the similarity between human and mouse MDP–cMOPs. Taken together, these findings suggest that MDPs are likely present in the tumor microenvironment of human cancers and may be committed to an immunosuppressive phenotype when located in tumors, but not in the blood, mirroring their behavior in murine tumors.

### Inhibition of monocytic-dendritic progenitors and common monocytic progenitors recruitment and differentiation into immunosuppressive monocytic cells sensitizes tumors otherwise resistant to immunotherapy

**Figure 5:**
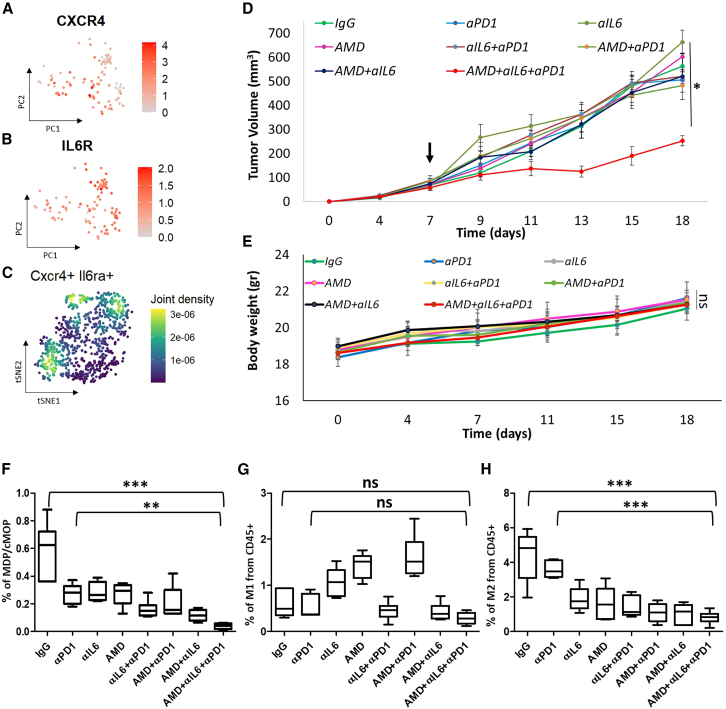
CXCR4 and IL6 inhibition improves the therapeutic outcome of 4T1 immunotherapy resistant tumors (A and B) Binned, normalized expression of CXCR4 (A) and IL6R (B) genes in human cells (as shown in Figure 4) is presented. (C) Binned, normalized co-expression of CXCR4 and IL6Ra in the mouse data. (D) 4T1p tumors implanted in BALB/c mice (n = 5–6 mice/group) were treated with anti-PD1, anti-IL6, AMD3100, or their combination thereof, using the schedule described in Figure S18A. A spider plot is available in Figure S18B. Treatment initiation is marked in a black arrow. Tumor growth was assessed regularly. (E) Body weight (in grams) of mice monitored regularly throughout the experiment. (F–H) When the tumor from D reached endpoint, tumors were removed and prepared as single-cell suspensions. Samples were analyzed for MDPs (F), M1-like (G), and M2-like (H) macrophages. Data are presented as mean ± standard deviation (SD). Statistical significance was assessed using one way ANOVA followed by Tukey post-test. Only comparisons of interest are shown. ns, non-significant. Significant *p* values are shown as ∗*p* < 0.05, ∗∗*p* < 0.01 and ∗∗∗*p* < 0.001.

Our preclinical findings in mice and validation in human data suggest that MDP-cMOPs accumulating in responding tumors may play a significant role in modulating the tumor microenvironment toward immunosuppression following immunotherapy. We postulate, therefore, that blocking MDP-cMOPs recruitment and/or their differentiation in the tumor microenvironment can improve the outcome of therapy-resistant tumors by inhibiting some of the sources of immunosuppressive cells. To test this, we sought two different strategies that impact MDP-cMOPs. First, we sought to inhibit their recruitment to tumors. We found that CXCR4, a receptor for the chemokine SDF-1, is highly expressed by MDP-cMOPs, especially those found in tumors (Figures 4B, 5A, and S17A). This could explain their recruitment to the tumor microenvironment, in particular when SDF-1 is highly expressed by cancer cells.^45^ Thus, disrupting the SDF-1-CXCR4 axis may inhibit the recruitment of MDP-cMOPs, among other immune cells, to the tumor microenvironment. Second, our previous study revealed that IL-6R is expressed by MDPs, and that blocking its signaling inhibits the differentiation of MDPs into immunosuppressive macrophages.^13^ Indeed, we found that IL-6R is also expressed by MDPs and their committed cells (Figures 5B and S17B). Therefore, we postulate that the inhibition of IL-6-IL-6R in tumors will block the differentiation of MDP-cMOPs into immunosuppressive macrophages, further increasing the likelihood of response to immunotherapy. Indeed, IL-6R and CXCR4 positive cells are found among the DA cells of sensitive tumors (Figure 5C), demonstrating their ability to differentiate into immunosuppressive macrophages. Using these two strategies, we assessed the therapeutic activity of anti-PD1 in combination with AMD3100, a small molecule antagonizing CXCR4, and anti-IL6 neutralizing antibodies, on immunotherapy-resistant triple negative 4T1 breast cancer. The treatment protocol is described in Figure S18A. Tumor growth was assessed over time, and tumors were removed at the endpoint to analyze MDP-cMOPs and macrophages. We found that 4T1 tumors treated with the combination of anti-PD1, AMD3100, and anti-IL6 exhibited remarkable therapeutic activity compared to all other drug combinations tested, including AMD3100 and anti-IL6 or other combinations thereof (Figures 5D and S18B). This combination did not result in major toxicities, as assessed by body weight measurements, and no clinical signs of toxic side effects were observed in any of the treatment groups (Figure 5E). At the experimental endpoint, tumors from the triple combination group showed significantly lower levels of MDP–cMOPs compared to IgG- and anti-PD1-treated tumors (Figure 5F). While the bar graphs display all treatment groups, statistical significance was specifically observed in comparisons between IgG, anti-PD1, and the triple combination. Notably, MDP–cMOP levels were low even in the combination group, suggesting that these cells failed to accumulate in these tumors, and not because they differentiate into immunosuppressive cells (Figures 5G and 5H). Indeed, the frequency of immunosuppressive (M2-like) macrophages was substantially lower in the three-drug combination, whereas in the anti-PD1 or IgG-treated groups, their levels were significantly higher. In contrast, M1-like macrophages did not display a significant difference between the groups (Figures 5G and 5H). These results suggest that MDP-cMOPs may play a role in facilitating resistance to immunotherapy. This may occur through their differentiation into immunosuppressive monocytic cells. These findings suggest that inhibiting MDP-cMOP recruitment and/or differentiation enhances anti-tumor immunity and improves anti-PD1 therapy.

## Discussion

Resistance to immunotherapy is a major obstacle and different mechanisms have been proposed to overcome it. Immunosuppressive cells such as MDSCs and subsets of macrophages have been shown to support immunotherapy resistance.^2^^,^^19^ The generation, differentiation, and expansion of the immunosuppressive myeloid cells from myeloid progenitor cells are mediated in part by the release of pro-inflammatory cytokines including GM-CSF, TNF-α, and IL-6.^12^ Our data demonstrate that these cytokines are highly expressed in sensitive tumors compared to resistant ones, and could therefore explain the induced differentiation of IMCs into immunosuppressive cells, thus supporting tumor resistance. While MDSCs are known to support therapy resistance, our data did not focus specifically on these cells. MDSCs in mice are typically defined by the co-expression of GR-1 and CD11b. In contrast, MDPs and cMOPs, which we found in higher abundance in ICI-sensitive tumors compared to resistant ones, lack CD11b expression but retain Ly6C expression, a component of the GR-1 protein complex.^21^ This supports their classification as IMCs rather than MDSCs. Moreover, a study in human gastric cancer identified a CD45^+^CD33^low^CD11b^dim^ MDSC subset capable of suppressing CD8^+^ T cell function via the IL-6/IL-8–arginase I axis, which also expressed CD66b.^46^ In our dataset, neither MDPs nor their committed progeny express CD66b (data not shown), further supporting that these populations are distinct from MDSCs and instead represent IMCs.

Using single-cell RNA-sequencing (scRNA-seq) from breast carcinoma, we found that MDPs and cMOPs are rarely found within tumors. Specifically, their frequency is higher in tumors responding to ICI therapy, whereas in resistant tumors, it is lower and inversely correlated to immunosuppressive macrophages. Our results suggest that in both humans and mice, MDP-cMOPs are pre-committed to immunosuppressive monocytes and, therefore, can explain therapy resistance. To overcome this, we demonstrated that blocking factors contributing to the recruitment of MDP-cMOPs into tumors and/or inhibiting their differentiation into immunosuppressive cells substantially improve immunotherapy efficacy in ICI-resistant tumors. Thus, our study provides a comprehensive analysis of the role of MDP-cMOPs in the tumor microenvironment and their potential impact on immunotherapy outcomes.

Our study focused on validating the presence of MDPs in human breast cancer and additional datasets, demonstrating their relevance beyond the 4T1 murine cancer model. While we did not extensively explore the underlying mechanisms or broader patterns of MDP differentiation in this study, emerging evidence suggests that MDP–cMOPs play a role in shaping the tumor microenvironment across different cancer types, further supporting an immunosuppressive microenvironment which contribute to metastasis.^13^ Future research integrating additional datasets and functional studies will be essential to determine the generalizability of our findings and to elucidate the specific roles of MDP–cMOPs in diverse tumor contexts.

Using RNA velocity, we showed that MDP-cMOPs may serve as the source of immunosuppressive monocytic cells accumulating in ICI-resistant tumors, results that have also been indirectly confirmed by flow cytometry using *in vivo* tumor model and directly confirmed through *in vitro* experiments. We therefore speculate that ICI therapy prompts the recruitment of MDPs to the treated tumor site, where they have no immunomodulatory function, but serve as a reservoir of cells poised to differentiate into immunosuppressive entities, further supporting therapy resistance. Interestingly, while MDPs have the capacity to differentiate into both monocytic and dendritic cells,^11^ our murine data fail to detect the pattern of MDP differentiation into dendritic cells. This absence may be attributed to the isolation of GR-1^+^ cells in the mouse dataset, which could have excluded early dendritic cell progenitors from the analysis. Furthermore, while the integration of human and mouse data strengthens the translational relevance of our findings, species-specific differences in immune cell phenotypes, marker expression, and lineage trajectories may limit direct comparability. For instance, MDPs in human tumors displayed potential differentiation into both cMOPs and CDPs, whereas in mice, MDPs predominantly committed to the monocytic lineage. These discrepancies highlight the need for cautious interpretation and further validation using functional assays in human systems. Nonetheless, merging human data from two distinct datasets of patients with breast cancer, as well as data from colorectal and lung cancers reveal that MDPs can differentiate into cMOP, cells that have already been committed to the monocytic arm^11^ or into CDPs, with the latter pathway leading to dendritic cell differentiation.^47^ We showed that MDP differentiation to CDPs predominantly resides within peripheral blood while MDPs in tumors are more likely to differentiate into cMOP. These results imply that MDPs within tumors are perhaps already committed to becoming immunosuppressive monocytes, as demonstrated in the murine data.

The identification of MDP-cMOPs within the tumor microenvironment suggests that tumors exploit their presence as a reservoir of cells that promote cancer cell survival. The existence and role of HSPCs within the tumor microenvironment remain incompletely understood, although evidence suggests their multifaceted contributions to tumor progression, even when located within the bone marrow or peripheral blood.^48^ For instance, increased levels of circulating HSPCs have been correlated with decreased survival in patients with solid tumors.^17^ Specifically, HSPCs were identified within the glioblastoma tumor microenvironment, further promoting glioblastoma cell proliferation and secreting pro-metastatic factors such as IL-6 and CCL2, alongside increased PDL1 expression by cancer cells.^17^ Similarly, in another study, it has been shown that GMPs migrate to the tumor and contribute to the generation of immunosuppressive microenvironment in 4T1 tumors.^18^ In the present study, we establish the presence of MDP-cMOPs within the treated tumor microenvironment, specifically in tumors that are sensitive to immunotherapy. Upon differentiation, these MDP-cMOPs confer greater aggressiveness to tumors and facilitate the establishment of a pro-tumorigenic microenvironment. We found that sensitive tumors exhibit lower levels of immunosuppressive monocytes in contrast to higher levels of MDP-cMOPs. Nevertheless, in tumors that undergo re-growth, the balance between MDP-cMOPs and immunosuppressive cells is inverted, further implying that they may contribute to immunotherapy resistance. Thus, evaluating MDP–cMOP levels in tumors may provide a potential prognostic biomarker for immunotherapy, as these cells can differentiate into immunosuppressive macrophages upon ICI therapy. Importantly, MDP–cMOP levels fluctuated when additional therapies were introduced that interfered with their accumulation and/or differentiation, as demonstrated with AMD3100 and anti-IL6 treatments. Overall, understanding the interplay between cancer cells and MDPs in ICI-treated tumors represents an important avenue for future research.

To counteract the emergence of resistance associated with MDP accumulation in tumors, we undertook two distinct strategies for MDP elimination: (a) impeding their recruitment to the immunotherapy-treated tumor microenvironment, and (b) obstructing their differentiation into immunosuppressive entities. Our comprehensive analysis highlighted CXCR4 as exhibiting high expression levels within the spectrum of immune cell populations, and mostly in MDPs. Given that CXCR4 acts as the receptor for SDF-1, a potent chemokine integral to tumor progression and growth,^45^ we employed AMD3100, a well-established CXCR4 antagonist widely used for HSPC mobilization from the bone marrow.^49^ Prior studies have demonstrated the anti-tumorigenic effects of AMD3100 in synergy with various therapies.^50^^,^^51^ A recent preclinical study has shown that combining immunotherapy with CXCR4 blockade in the context of triple-negative breast cancer leads to enhanced therapeutic efficacy.^51^ This combined approach reduces the infiltration of immunosuppressive immune cells to tumors while promoting T lymphocyte infiltration, partially attributed to the mitigation of the desmoplastic nature of tumors.^51^ In light of these findings, the therapeutic potential of CXCR4 inhibition to curtail MDP-cMOP recruitment to tumors appeared promising. Indeed, our observations confirmed that concurrent CXCR4 blockade and ICI therapy resulted in diminished MDP-cMOP presence within tumors. While alternate anti-tumor immune mechanisms related to AMD3100 remain plausible, the notable reduction of MDP-cMOPs within the tumor microenvironment implies its potential role in mitigating MDPs as a “ticking bomb”.

In addition to MDP recruitment, we directed our efforts toward restricting the differentiation of MDPs into immunosuppressive entities. A previous study revealed high expression of IL-6R in MDPs and demonstrated that metastatic tumors exhibiting elevated IL-6 levels prompted the differentiation of MDPs into M2-like macrophages.^13^ Inhibiting the IL6-IL6R interaction within MDPs led to a reduced metastatic burden, underscoring the capacity to restrain their differentiation into immunosuppressive cells.^13^ Building upon these findings, we harnessed this approach to impede MDP differentiation. Notably, the synergy of anti-PD1, anti-IL-6, and AMD3100 resulted in attenuated tumor growth, even in intrinsically immunotherapy-resistant tumors. These favorable outcomes were concomitant with decreased MDP-cMOP presence and a reduction in M2-like immunosuppressive macrophages.

With respect to eliminating MDP recruitment and differentiation in tumors in order to improve immunotherapy outcomes, a multitude of studies have documented additional mechanisms through which anti-IL-6 and CXCR4 antagonists affect immunotherapy outcomes. For instance, the neutralization of IL-6 in combination with immunotherapy has been shown to confer benefits in diverse preclinical models.^3^^,^^13^ Similarly, CXCR4 antagonism in synergy with anti-cancer agents has been demonstrated to enhance therapeutic efficacy by inhibiting MDSC recruitment to tumors.^50^ Thus, the efficacy of our combination therapy may extend beyond restricting MDP recruitment and differentiation within tumors. Nonetheless, the escalating integration of immunotherapy within neoadjuvant treatment strategies, particularly in the context of triple-negative breast cancer, highlights the necessity to enhance response rates and overall outcomes. This is of utmost importance, particularly in scenarios where surgical resection constitutes a key facet of the therapeutic protocol. By employing our combined therapeutic approach, the risk of impeding tumor debulking can be mitigated. This approach also helps prevent the need for changes to treatment regimens prior to surgery. Notably, based on our previous studies^3^^,^^13^^,^^45^ and the present study, this combined therapeutic strategy exhibits the potential to arrest tumor aggressiveness and reduce the risk of metastasis. Consequently, rigorous evaluation of the combined therapeutic agents can be of great benefit, especially when considering the individual FDA approvals of each drug for diverse indications.

### Limitations of the study

The current study has several limitations. While our combined therapeutic regimen exhibits potency in diminishing MDP numbers and fostering anti-tumor activity, its exclusive impact on MDPs is yet to be fully elucidated. In addition, it should be acknowledged that the inhibitors used (both for CXCR4 and IL6R inhibitors) may have off-target effects that could contribute to the observed outcomes. Therefore, further studies are required to separate these effects from their specific roles in MDP recruitment and differentiation. Furthermore, in our study, we were unable to demonstrate the direct differentiation of MDPs into M2-like macrophages *in vivo*. Although we attempted lineage-tracing experiments using GFP labeling to monitor the differentiation of MDPs into macrophages, the extremely low number of GFP-positive macrophages hindered reliable detection. Nonetheless, we were able to demonstrate *in vitro* that MDP-cMOPs differentiate into immunosuppressive macrophages in the presence of conditioned medium from 4T1p cancer cells, an effect associated with IL-6. These findings are consistent with a previous study showing that IL-6 expressed by metastatic tumors promotes MDP differentiation into immunosuppressive macrophages.^13^ Altogether, our results provide further support for the role of ICI-resistant tumors in driving the differentiation of MDPs into M2-like monocytes. However, additional studies are needed to validate this process *in vivo*.

## Resource availability

### Lead contact

Further information and requests for resources and reagents should be directed to and will be fulfilled by the lead contact, Yuval Shaked (yshaked@technion.ac.il).

### Materials availability

This study did not generate new unique reagents. All materials used in this study are commercially available or can be provided by the lead contact upon reasonable request.

### Data and code availability

• The scRNA-seq data generated in this study have been deposited at the Gene Expression Omnibus (GEO), and are publicly available as of the date of publication. Accession numbers are listed in the key resources table.
• This study did not generate original code.
• Any additional information required is available from the corresponding authors upon request.

## Acknowledgments

This work was supported by grants from the 10.13039/501100003977Israel Science Foundation (grant no. 194/18 to Y.S. and grant no. 3614/19 to K.Y.). We are grateful to the Rubinstein Scholarship Foundation for supporting S.L., and to the Adrian the Rothschild Scholarship Foundation for supporting M.B.

## Author contributions

Conception and design: S.L., K.Y., and Y.S. Acquisition of data: S.L., M.B., B.M., C.B., and Z.R. Analysis and interpretation of data: S.L., Z.R., K.Y., and Y.S. Writing, review, and/or revision of the article: S.L., K.Y., and Y.S. Study supervision: K.Y. and Y.S.

## Declaration of interests

The Animal Care and Use Committees of the Technion (Haifa, Israel) approved all animal studies and experimental protocols. All authors have read the article and approved its content. The dataset will be available online upon publication. The authors declare that no conflict of interest. The authors declare that the graphical abstract was created with the aid of BioRender.

## STAR★Methods

### Key resources table

**Table:** 

REAGENT or RESOURCE	SOURCE	IDENTIFIER
**Antibodies**		

Anti-mouse Ly-6G/Ly-6C (Gr-1)- (Clone RB6-8C5)	BioLegend	Cat# 108402, RRID: AB_313366
Anti-mouse Ly-6A/E (Sca-1)- (Clone D7)	BioLegend	Cat# 108139, RRID: AB_2565957
Anti-mouse CD117 (c-kit)- (Clone ACK2)	BioLegend	Cat# 105802, RRID: AB_313210
Anti-mouse CD34- (Clone SA376A4)	BioLegend	Cat# 152203, RRID: AB_2629647
Anti-mouse CD115 (CSF-1R)- (Clone AFS98)	BioLegend	Cat# 135523, RRID: AB_2566459
Anti-mouse CD135 (Flt3)- (Clone A2F10)	BioLegend	Cat# 135311, RRID: AB_2107049
Anti-mouse CD45- (Clone 30-F11)	BioLegend	Cat# 103127, RRID: AB_493714
Anti-mouse CD11b- (Clone M1/70)	BioLegend	Cat# 101229, RRID: AB_2129375
Anti-mouse F4/80- (Clone BM8)	BioLegend	Cat# 123109, RRID: AB_893498
Anti-mouse CD206 (MMR)- (Clone C068C2)	BioLegend	Cat# 141717, RRID: AB_2562232
Anti-mouse CD11c- (Clone N418)	BioLegend	Cat# 117323, RRID: AB_830646
Anti-mouse I-A/I-E (MHC class II)- (Clone M5/114.15.2)	BioLegend	Cat# 107635, RRID: AB_2561397
Anti-PD-1 (Clone RMP1-14-CP151)	BioXcell	Cat# CP151, RRID: AB_2927525
Anti-IL-6 (Clone MP5-20F3)	BioXcell	Cat# BE0046, RRID: AB_1107709
Plerixafor (AMD3100)	APExBIO	Cat# A2025-100
IgG	BioXcell	Cat# CP150, RRID: AB_2927524

**Chemicals, peptides, and recombinant proteins**		

1-methyl-3-nitro-1-nitrosoguanidine	Apollo Scientific	Cat# OR301388
Dulbecco’s Modified Eagle’s Medium - high glucose	Sigma	Cat# D5796
Fetal Bovine Serum	Biological Industries	Cat# 10270-106
L-Glutamine Solution	Biological Industries	Cat# 03-020-1B
Sodium Pyruvate Solution	Biological Industries	Cat# 03-042-1B
Penicillin-Streptomycin	Sigma	Cat# P4333-100ML

**Critical commercial assays**		

Proteome Profiler Mouse XL Cytokine Array	R&D Systems	Cat# ARY028
EasySep™ Mouse PE Positive Selection Kit II	Stemcell Technologies	Cat# 17666

**Experimental models: Cell lines**		

4T1 (CRL-25390)	ATCC	N/A
4T1 mutagenized	This paper	N/A

**Experimental models: Organisms/strains**		

BALB/c mice	Envigo	Cat# 162
C57Bl/6 mice	Envigo	Cat# 057

**Deposited data**		

Mouse single-cell RNA sequencing data of GR-1 cells	This paper	GEO: GSE298984
Mouse single-cell RNA sequencing data of bone marrow cells	Tabula Muris et al.^52^	GEO: GSE132042
Human single-cell RNA sequencing data of breast cancer	Zhang et al.^53^	GEO: GSE123814
Human single-cell RNA sequencing data of breast cancer	Bassez et al.^54^	lambrechtslab - Single cell
Human single-cell RNA sequencing data of colon cancer	Pelka et al.^43^	GEO: GSE178341
Human single-cell RNA sequencing data of lung cancer	Salcher et al.^44^	https://doi.org/10.5281/zenodo.6411867

**Software and algorithms**		

CellRanger [v5.0.1]	10x Genomics	https://www.10xgenomics.com/support/software/cell-ranger/latest
R [v4.2.2]	R Core Team	https://www.r-project.org/
Seurat [v4.3.0]	Stuart et al.^55^	https://satijalab.org/seurat/
scDblFinder [v1.12.0]	Germain et al.^56^	https://github.com/plger/scDblFinder
SCINA [v1.2.0]	Zhang et al.^53^	https://github.com/jcao89757/SCINA
DASeq [v1.0.0]	Zhao et al.^26^	https://github.com/KlugerLab/DAseq
wBoot [v1.0.3]	Neil A. Weiss	https://github.com/cran/wBoot/tree/master
velocyto [v0.17]	La Manno et al.^25^	velocyto | Estimating RNA velocity in single cell RNA sequencing datasets
scVelo [v0.2.5]	Bergen et al.^57^	https://scvelo.readthedocs.io/en/stable/
clusterProfiler [v4.2.6]	Yu et al.^58^	https://guangchuangyu.github.io/software/clusterProfiler/
MsigDB [v7.5.1]	Liberzon et al.^59^	https://github.com/mw201608/msigdb
scVI [v1.2.2]	Gayoso et al.^60^	https://scvi-tools.org/
SingleR [v2.0.0]	Aran et al.^61^	https://github.com/dviraran/SingleR
Scanpy [v1.10.4]	Wolf et al.^62^	https://scanpy.readthedocs.io/en/stable/
Slingshot [v2.6.0]	Street et al.^42^	https://github.com/kstreet13/slingshot

**Software**		

FlowJo [v10]	BD Bioscience	https://www.flowjo.com/
Prism [v5]	GraphPad	https://www.graphpad.com/features
Python [v3.10.8]	Python Software Foundation	https://www.python.org/

### Experimental model and study participant details

#### Animals

Female BALB/c (8-10 weeks of age) were purchased from Envigo, Israel. The use of animals and experimental protocols were approved by the Animal Care and Use Committee of the Technion (ethic approval number: IL1200822). Female mice were chosen due to the use of a breast cancer model. All mice were maintained under specific pathogen-free conditions in the animal facility.

#### Cell line and cell culture

The 4T1 murine breast carcinoma cell line was obtained from the American Type Culture Collection (ATCC, Manassas, VA, USA) and used within six months of thawing from the original authenticated stock. Cells were routinely tested and confirmed to be mycoplasma-free. To generate cancer cells sensitive to ICI therapy, we followed a previously published protocol.^9^^,^^15^ Briefly, 4T1 cells—initially resistant to ICI therapy (designated 4T1p)—were treated with 1-methyl-3-nitro-1-nitrosoguanidine (MNNG) for 2 hours. The cells were then washed with PBS, followed by the addition of fresh growth medium. After a recovery period of 5 days, a multiclonal population of mutagenized cells (designated 4T1m) was established. All cells were cultured at 37°C in 5% CO_2_ in Dulbecco’s Modified Eagle Medium (DMEM, Sigma-Aldrich, Rehovot, Israel) supplemented with 10% fetal calf serum (FCS, Biological Industries, Israel), 1% L-glutamine, 1% sodium pyruvate, and 1% penicillin-streptomycin-neomycin solution (Biological Industries, Israel).

#### Murine tumor models and treatments

4T1p and 4T1m cells (5x10^5^/50μL in serum-free medium) were orthotopically injected into the mammary fat pad of 8–10-week-old female BALB/c mice. Mice were randomized before treatment initiation, and the analysis of the results was performed blindly. In all experiments, when tumors reached ∼50 mm^3^ on average, mice were treated with anti-mouse PD-1 antibody (clone RMP1-14, BioXCell or Ichorbio) , given twice a week at a dose of 100μg/mouse for up to a 2-week period. In some experiments, mice were treated with anti-IL-6 antibodies (20mg/kg, MP5-20F3 clone; BioXCell, Lebanon, NH, USA) or IgG control antibodies (20mg/kg), twice a week.^3^^,^^13^ AMD3100, a small-molecule CXCR4 antagonist (APExBIO; 5 mg/kg/day) was injected subcutaneously.^45^ Tumor volumes were measured regularly using a Vernier caliper and volume was calculated by using the formula width^2^×length×0.5. Body weight measurements were taken using a scale to ensure mouse welfare, and mice were routinely monitored for clinical signs and behavior.

### Method details

#### Cytokine array

Cells derived from 4T1m and 4T1p tumors were cultured in serum-free medium at a density of 10^6^ cells/ml for 24 hours to generate conditioned medium. The conditioned medium was then applied on a Proteome Profiler Mouse XL Cytokine Array (ARY028, R&D Systems, MN), following the manufacturer’s instructions. Protein expression was quantified via densitometry. Differences in the relative expression of proteins between the 4T1m and 4T1p tumors were calculated and are presented as log2 fold change.

#### Evaluation of MDP-cMOP differentiation towards M2 macrophages

To assess the differentiation pattern of MDP-cMOPs in response to tumor-derived factors, 4T1p and 4T1m cells (1×10^6^/mL) were cultured for 24 hours in serum-free RPMI to generate conditioned medium (CM). MDPs (1.5×10^4^ cells/mL), isolated from the bone marrow of naïve C57Bl/6 mice using flow cytometry, were then cultured in serum-free medium containing 75% CM of cancer cells, in the presence or absence of anti-IL-6 neutralizing antibodies (25 ng/mL). After two days of incubation at 37°C in a humidified atmosphere with 5% CO_2_, cells were analyzed by flow cytometry for M2 macrophage surface markers.

#### Murine single-cell RNA sequencing (scRNA-seq) on GR-1+ cells

The evaluation of GR-1^+^ myeloid cells in 4T1p and 4T1m tumors after two weeks of anti-PD1 therapy was performed using scRNA-seq. Briefly, tumors were enzymatically and mechanically dissociated into single-cell suspensions. Enzymatic digestion was carried out using collagenase IV and DNase I at 37°C for 45 minutes, followed by mechanical disruption.^63^ GR-1^+^ cells were then isolated by positive selection using the EasySep Mouse PE Positive Selection Kit (BioLegend), according to the manufacturer’s instructions. Purified GR-1^+^ cells were washed in PBS containing 0.04% BSA to minimize cell clumping and resuspended at a concentration of 1,000 cells/μL in PBS. RNA was immediately extracted, and cells were processed using the 10x Genomics Chromium platform for single-cell sequencing. Library preparation was performed using the Chromium Single Cell 3′ Library & Gel Bead Kit v3 (10x Genomics).

#### Flow cytometry acquisition and analysis

To validate cell subpopulations, tumors underwent single-cell suspension,^63^ and cells were immunostained with different antibodies based on markers indicated in Table S5. Figures S9 and S10 represent the gating strategy for the detection of MDP-cMOP cells and macrophage subpopulations, respectively. All monoclonal antibodies were purchased from BioLegend and were used in accordance with the manufacturer’s instructions. At least 300,000 events were acquired using a BD LSRFortessa flow cytometer and analyzed with FlowJo [v 10.6.2] software (FlowJo, Ashland, Oregon, USA).

### Quantification and statistical analysis

#### Pre-processing of scRNA-seq data

The Seurat pipeline [v4.3.0]^55^ was used for downstream analysis. Data were read into R [v4.2.2], and gene expression cutoffs were applied to retain cells with at least 250 and at most 5,000 detected genes per cell. These thresholds were chosen based on prior experience and published guidelines to ensure the inclusion of high-quality cells while excluding potential outliers. Additionally, cells with over 12.5% mitochondrial gene content were excluded to eliminate likely dead cells and a maximum threshold of 27,000 UMIs per cell was applied. The scDblFinder [v1.12.0]^56^ was then used to remove doublets. Genes Gm42418 and AY036118 were also removed, as they overlap with the rRNA element Rn45s and represent rRNA contamination.^64^ We also set PTPRC >0 threshold to remove non-immune related cells. Finally, the ‘SCTransform’ [v3] function^55^ was applied to normalize and scale the data, selecting 3000 variable features and linearly regressing out mitochondrial genes for downstream analyses.

#### Processing of scRNA-seq data

Raw, Illumina base calls (BCLs) were demultiplexed and the resulting FASTQ files were aligned to the mm10 (GRCm38, Ensembl 93) murine reference genome. Data were normalized for sequencing depth using 10x Genomics CellRanger [v5.0.1] to generate expression matrices. Reads (80.5-88.4%) mapped to the transcriptome across all samples. A median of 2,763 unique molecular identifiers (UMIs) per cell for 4T1p and 2,066 UMIs per cell for 4T1m were observed, respectively. Please see STAR Methods for computational data analysis of the scRNA-seq.

#### Dimensionality reduction and cell type classification

Dimensionality reduction was performed with principal component analysis (PCA) followed by t-SNE projection. Unsupervised clustering was conducted with the shared nearest neighbor (SNN), using the first 11 principal components and the Seurat functions ‘FindNeighbors’ and ‘FindClusters’.^55^ Subsequently, two lymphocytic clusters were excluded due to the high expression of B cell markers (Cd79a, Cd79b, Ms4a1) and T cell markers (Ms4a4b, Cd3g, Cd3e). For cell type identification, we utilized the Semi-supervised Category Identification and Assignment (SCINA) method,^65^ using a gene set specific to GR-1+ myeloid subset markers. SCINA’s classification is based on stringent marker expression criteria, and cells that did not exhibit sufficient signature expression were categorized as 'unknown' to maintain high-confidence annotations. The presence of 'unknown' cells reflects the method’s conservative assignment approach rather than misclassification. The gene set used for SCINA is shown in Figure 1E.

#### Differential abundance

Cellular neighborhoods with differential abundance (DA) between 4T1m and 4T1p were identified by DASeq [v1.0.0]^26^ using the principal components matrix (Figures 1A and 3A). In addition, the significance of cell subtypes proportions was carried out using the bias-corrected and accelerated (BCa) bootstrap method for independent two-sample comparisons, followed by one-sided hypothesis testing, conducted through wBoot package in R [v1.0.3] (Figure 2A).

#### RNA-velocity

To explore how cells transition between different states, we performed RNA velocity analysis. We first calculated the ratio of unspliced to spliced mRNAs for approximately 20,000 genes using velocyto [v0.17].^25^ Next, we used Python [v3.10.8] and the scVelo package [v0.2.5]^57^ to estimate each cell’s developmental progression (pseudotime) and the likely direction of state changes (RNA velocity). We also included CD11b expression as an additional marker of cell differentiation. These results were visualized by overlaying RNA velocity vectors, pseudotime scores, and CD11b expression on a t-SNE plot generated with Seurat.

#### Gene expression and gene set enrichment analysis

The Wilcoxon rank-sum test within the “FindAllMarkers” function was used to analyze all single- cell differential gene expressions (DEGs) across identified cell subsets. For the purpose of gene set enrichment analysis (GSEA), DEGs between the two groups were identified using the “FindMarkers” function with default parameters. To characterize MDP-cMOP cells, the DEGs for MDP-cMOP compared to all other cells were processed into Gene set enrichment analysis (GSEA)^66^ using clusterProfiler [v4.2.6].^58^ Gene-lists from the MsigDB [v7.5.1]^59^ (category = C8, cell type signature) were used as a reference. In addition, DEGs between differentially abundant cells of 4T1m and 4T1p were calculated in order to assess the functional changes between the tumors and their DA regions. Significantly enriched genes were defined by an adjusted p-value (FDR) threshold of <0.01 and a log2 fold-change threshold of >1 (using the Benjamini & Hochberg method).^67^ These thresholds were chosen based on extensive prior literature and our exploratory data analyses. The FDR threshold of <0.01 was implemented to rigorously control for false positives in the context of multiple comparisons, ensuring that only highly reliable changes were considered. Similarly, the log2 fold-change threshold of >1 was selected to focus on genes with expression changes that are likely to be biologically meaningful. The filtered gene list was then processed through the “enrichGO” function using the GO Biological Process ontology.

#### Batch-effect removal and data integration

We integrated MDPs from tumors and bone marrow (BM) using scVI, a conditional variational autoencoder-based method.^60^ Initially, we annotated a scRNA-seq atlas from the BM of non-tumor-bearing mice (Figure S8A).^52^ SingleR [v2.0.0]^61^ was then used to compare bone marrow cells to the ImmGen reference provided by celldex [v1.8.0], identifying low-quality cells labeled as “Unidentified” based on the “prunescores” function with default settings. Subsequently, MDPs from the bone marrow were merged with the MDP-cMOP cluster from the 4T1 tumors. The combined dataset (n=1244) was converted to Scanpy object^62^ and clustered using the top 2000 variable genes with the ‘seurat_v3’ method (Figure S8B). We then trained an scVI model on the counts following the approach described in,^60^ and visualized the integrated data using the scVI latent space (Figure S8B). Finally, we computed DEGs from the scVI normalized expression matrix via the Wilcoxon rank-sum test in Scanpy (Figure S8C).

#### Human scRNA-seq analysis of tumor and blood from cancer patients

We utilized two scRNA-seq datasets from the tumor and blood of breast cancer samples: Zhang et al.^53^ (GSE123814) and Bassez et al.^54^ (http://biokey.lambrechtslab.org). These studies included tumor and blood samples from patients with primary and metastatic breast cancer who received treatments involving immunotherapy, chemotherapy, or their combination thereof. Surface markers of hematopoietic progenitor cells were evaluated across the datasets. The processed data were imported into R [v4.2.2] and log-normalized using the Seurat package [v4.3.0].^68^ Cells were first subsetted to include only those with positive expression (>0) of both CD38 and CD117. The remaining cells from each dataset were merged, resulting in a final count of 38 blood cells and 56 tumor cells. The “SCTransform” [v3]^68^ function was then used for data normalization and scaling. Dimensionality reduction was performed with the first three principal components, as shown in PCA plot (Figures 4A and S11). No batch correction was observed in the data (Figure S11). Unsupervised clustering was performed with a SNN approach based on the first three PCs. Four clusters and their respective commitments were defined based on surface markers: b_MDP, t_MDP, t_cMOP, and CDP, identified in both blood and tumor samples, where b represents blood and t represents tumor. The CDP cluster was further subdivided into b_CDP and t_CDP. The surface markers used to define these clusters are listed in Table S5. DEGs between clusters were identified using the “FindAllMarkers” function with default parameters. Trajectory analysis was conducted using Slingshot [v2.6.0],^42^ with b_MDP cells defined as the root. Gene expression changes along the trajectory were assessed by ranking the 1,000 most variable genes and fitting a Generalized Additive Model (GAM) with a LOESS term for pseudotime.^69^ A heatmap displaying the top 50 genes that varied in expression over pseudotime was generated using the clusterExperiment [v2.18.2] package.^70^

In addition, scRNA-seq datasets of colorectal cancer^43^ (GSE178341) and lung cancer^44^ were analyzed to characterize the immune cell composition within tumors, with a particular focus on identifying MDPs and cMOPs.

#### Visualization of gene expression

Gene expression data was visualized using the “FeaturePlot” function from Seurat^55^ and the “plot_density” function from the Nebulosa [v1.8.0],^71^ which recovers gene expression signals through kernel density estimation. Additionally, scVelo tools^57^ were used to visualize the expression of CD11B.

#### Statistical analysis

All statistical tests for Flow Cytometry data were performed using GraphPad Prism [v5] software (La Jolla, CA, USA). Statistical pairwise comparisons were carried out using unpaired two-sample Mann-Whitney test, or by one-way ANOVA coupled with Tukey’s post-hoc HSD test when comparing more than two groups. At least 5 mice per group were used in order to reach statistical power considering a Gaussian distribution. The mice that exhibited pathological conditions unrelated to the experiment were excluded from the analyses. All experiments were performed in a randomized manner, when animals were distributed equally between groups before treatment was initiated. The *in vivo* experiments were repeated at least twice. Data are presented as mean ± standard deviation (SD). The significance of cell subtypes’ proportion in the scRAN-seq data, was carried out using bias-corrected and accelerated (BCa) bootstrap method for independent two-sample comparisons, followed by one-sided hypothesis testing, and conducted through wBoot package in R [v1.0.3]. *P*-values were set as ∗p <0.05; ∗∗p <0.01; and ∗∗∗p <0.001.

## Supplemental information

Document S1. Figures S1–S18 and Table S5

Table S1. Differentially expressed genes between granulocyte cluster cells that are abundant in 4T1m compared to 4T1p, related to Figure 1 The table lists the differentially expressed genes of granulocytic cells upregulated in 4T1m and downregulated in 4T1p. The genes are ordered based on significance.

Table S2. Enrichment analysis of the MDP-cMOP cluster compared to all other cells, related to Figure 1 The table lists the enriched pathways upregulated in MDP-cMOP cluster and downregulated in all other clusters in both 4T1m and 4T1p datasets. The enriched pathways are ordered based on significance.

Table S3. Differentially expressed genes upregulated in MDP-cMOP compared to MDSC, related to Figure 1 The table lists the differentially expressed genes upregulated in MDP-cMOP cluster and downregulated in MDSC cluster in both 4T1m and 4T1p datasets. The genes are ordered based on significance.

Table S4. Differentially expressed genes upregulated in MDP-cMOP compared to M0, related to Figure 1 The table lists the differentially expressed genes upregulated in MDP-cMOP cluster and downregulated in non-activated macrophage (M0) cluster, in both 4T1m and 4T1p datasets. The genes are ordered based on significance.

Table S6. Differentially expressed genes of monocytic cells between differential abundance regions in 4T1m-Mon and 4T1p-Mon, related to Figure 3 The table lists the differentially expressed genes of monocytes upregulated in 4T1m and downregulated in 4T1p. The genes are ordered based on significance.
