# Supplementary material for: Immature monocytic cells within tumors differentiate into immunosuppressive cells in resistant tumors to immunotherapy
Source: iScience. 2025 Jul 17;28(8):113141. doi: 10.1016/j.isci.2025.113141 (PMC12357114; doi:10.1016/j.isci.2025.113141)
Supplement: Document S1. Figures S1–S18 and Table S5 [file mmc1.pdf]

iScience, Volume *28*

Supplemental information

Immature monocytic cells within tumors differentiate into immunosuppressive cells in resistant tumors to immunotherapy

Sapir Levin, Madeleine Benguigui, Bar Manobla, Chen Buxbaum, Ziv Raviv, Keren Yizhak, and Yuval Shaked

Supplemental information online

Supplemental Figures

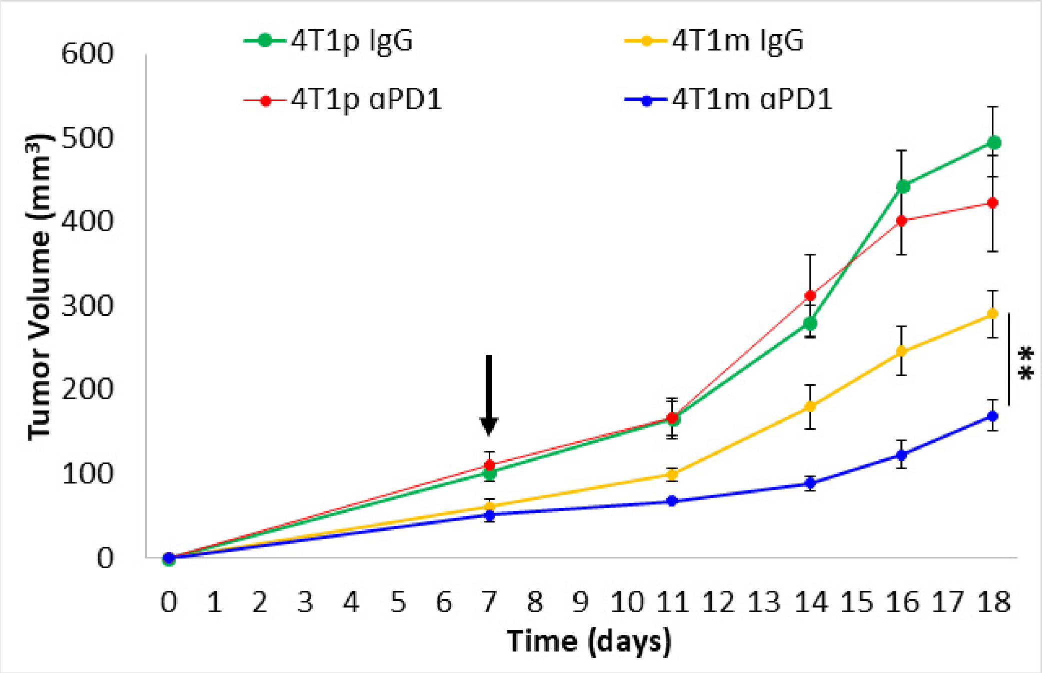

Figure S1: Tumor growth of 4T1m and 4T1p cell lines following treatment with anti-PD1, related to Figure 1. Eight to ten-week-old BALB/c female mice (n=5 mice/group) were implanted with 4T1p or 4T1m cancer cells (0.5×10^6 cells/mouse) into the mammary fat pad. After 7 days, when tumors reached ~50mm^3, treatment with anti-PD1 (100µg/mouse) or IgG control was initiated (black arrow), given twice a week for a two-week period. Tumor growth was assessed regularly. Data are presented as mean ± standard deviation (SD). Statistical significance for the endpoint was assessed using a t-test when comparing IgG control and anti-PD1 for each cell line. A significant p-value is shown as **p<0.01.

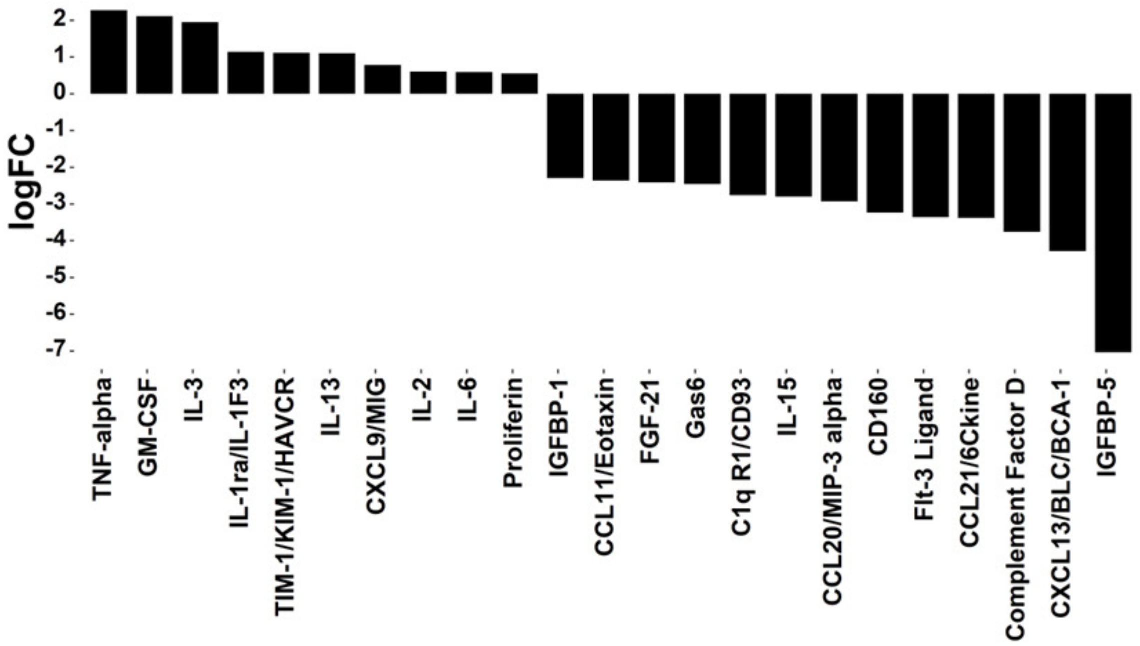

Figure S2: Differential expression of cytokines between 4T1m and 4T1p tumors, related to Figure 1. Conditioned medium from 4T1m and 4T1p tumor cultures was analyzed using a cytokine array to compare relative cytokine expression levels. The figure highlights selected cytokines with significantly altered expression, quantified as log2 fold changes (FC) of 4T1m over 4T1p groups.

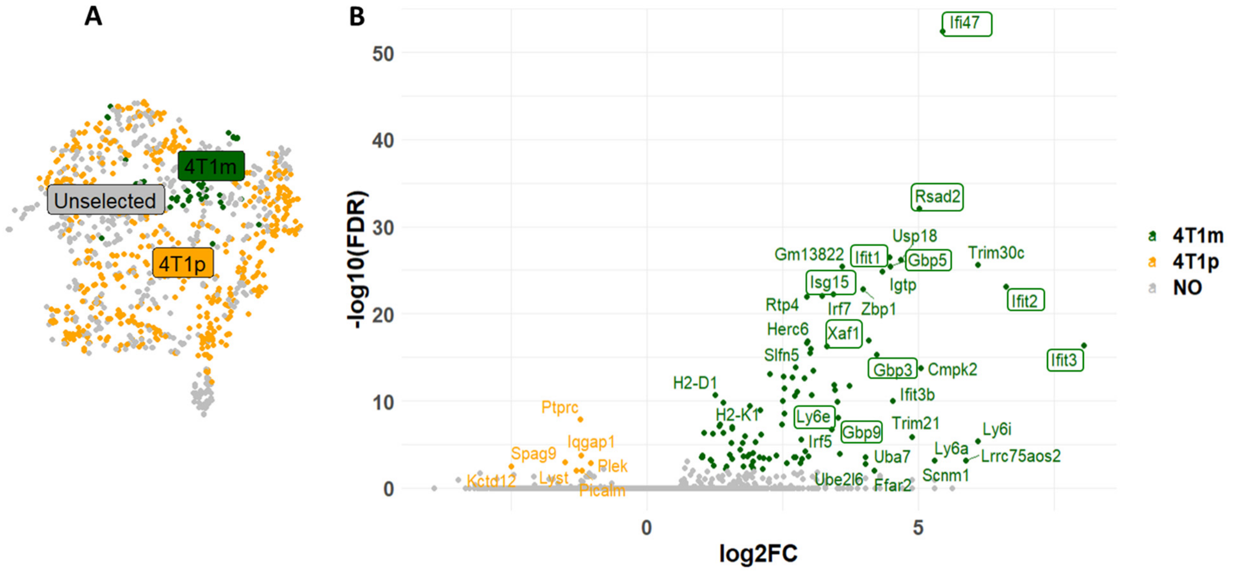

Figure S3: Differential abundance of the granulocytic subset between 4T1p and 4T1m tumors following anti-PD1 therapy, related to Figure 1. (A) A tSNE plot of 1,049 filtered, GR-1$^+$ granulocytic cells, with cells colored based on differential abundance scores between 4T1m (green) and 4T1p (orange) groups. (B) DEGs between 4T1m and 4T1p are presented in a volcano plot (FDR< 0.01, and log2 fold-change > 1 or log2 fold-change < -1). Notably, genes associated with Ly6E neutrophils are shown.

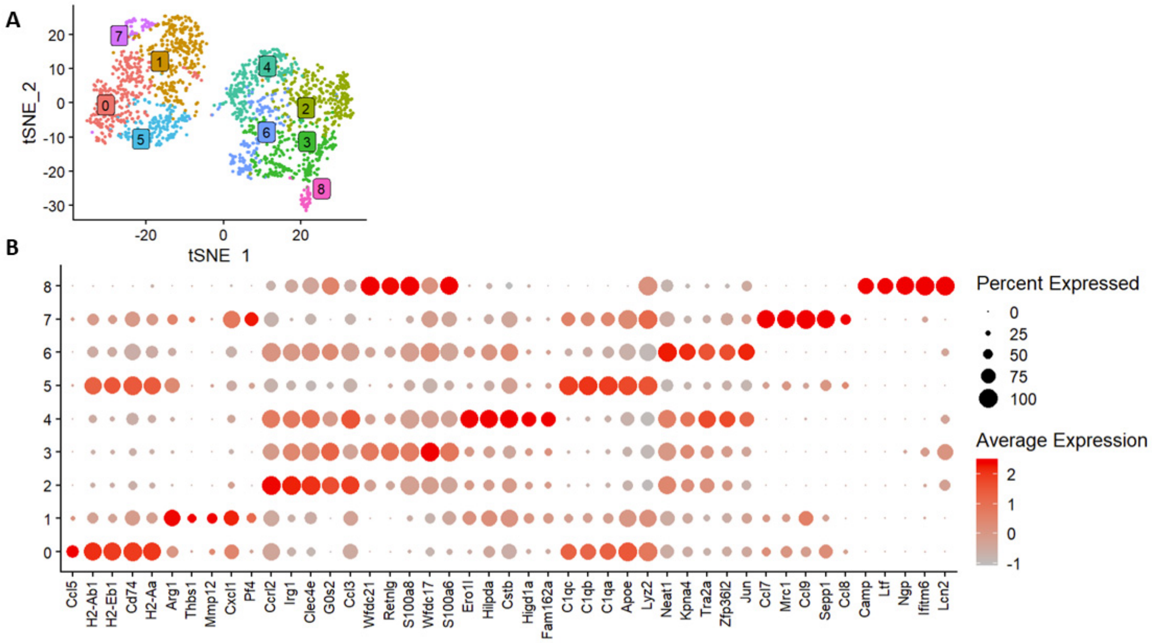

Figure S4: Unsupervised clustering of GR-1+ cells in 4T1m and 4T1p tumors following anti-PD1 therapy, related to Figure 1. (A) A tSNE plot of 9 different clusters is shown, displaying two major populations, monocytic and granulocytic cells, and nine different clusters (clusters 0-8). (B) The top 5 highly expressed genes in each cluster are shown, demonstrating similar clustering to that found using the semi-supervised approach. Notably, clusters 2 and 5 represent MDP-cMOP populations based on the expression of genes such as C1q and MHC class II gene family.

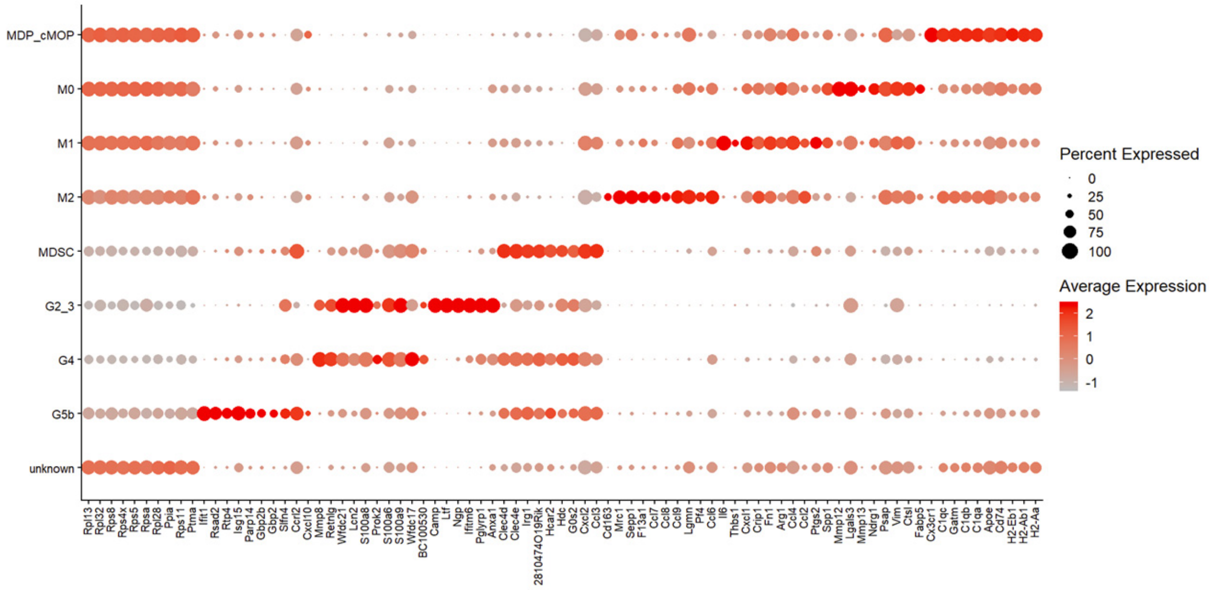

Figure S5: Gene expressions in different Gr-1+ clusters, related to Figure 1. Cell types were identified using a semi-supervised clustering method and DEGs between clusters were calculated using the Wilcoxon rank-sum test. The top 10 most highly expressed significant genes in each cluster are shown.

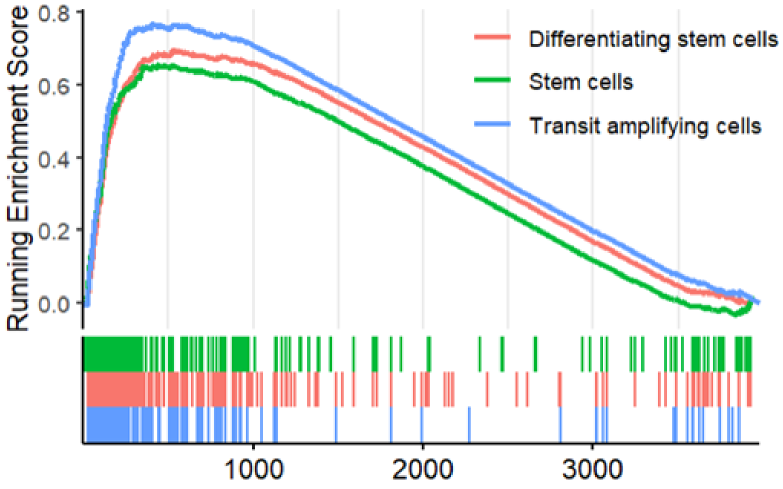

Figure S6: Enrichment analysis of MDP-cMOP cluster, related to Figure 1. Using Gene set enrichment analysis (GSEA) clusterProfiler [v4.2.6], and gene lists from the MsigDB[v7.5.1] (category = C8, cell type signature), the pathway enrichment of MDP-cMOP cluster was analyzed when compared to all monocytic clusters. Notably, the pathway enrichment demonstrated genes associated with stem cells, and differentiating stem cells, further supporting that MDP-cMOP cluster represents the immature state of monocytic cells.

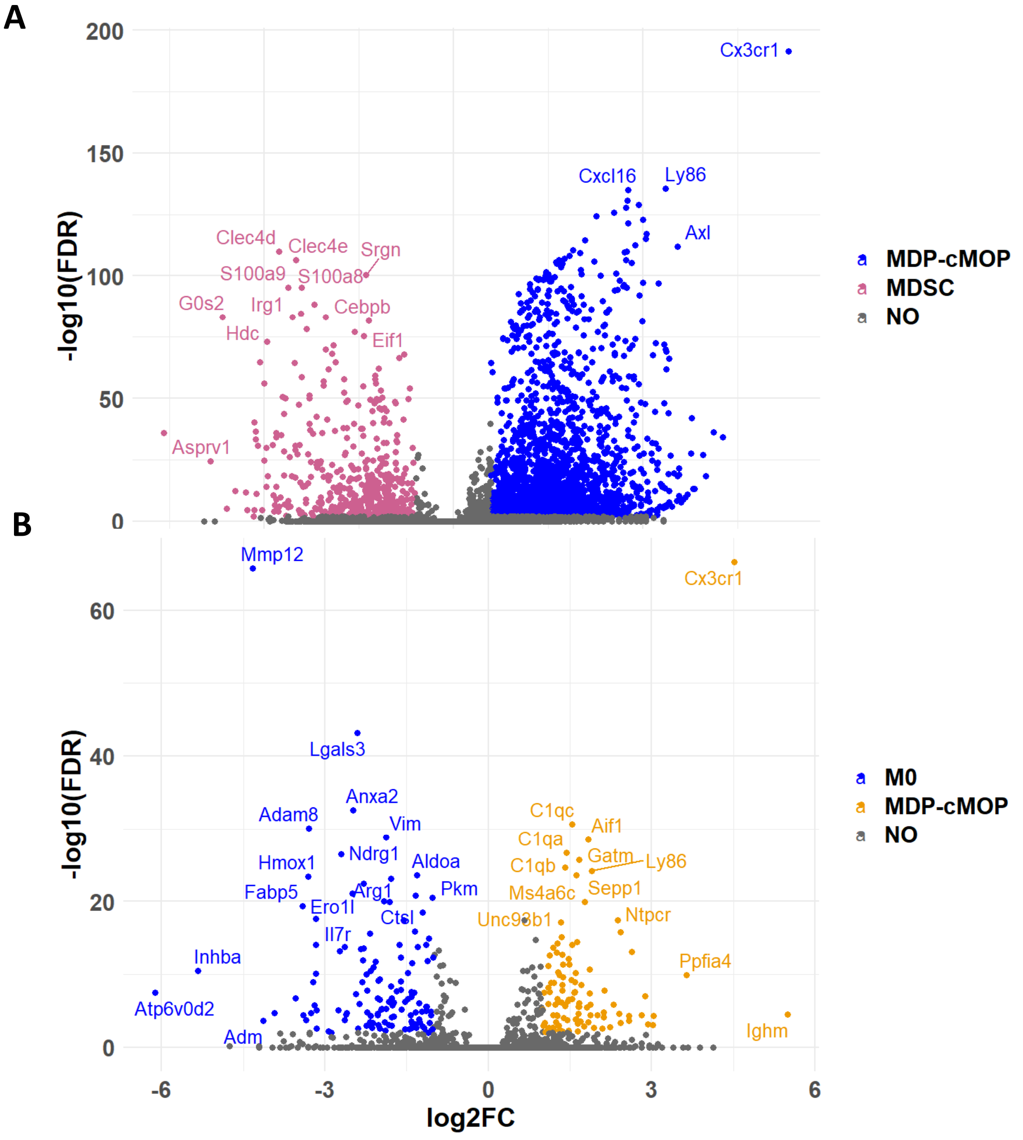

Figure S7: DEGs between MDP-cMOP and immature myeloid cells, related to Figure 1. (A) DEGs between MDP-cMOP and MDSCs, and (B) DEGs between MDP-cMOP and M0 macrophages, highlighting key MDP-cMOP marker genes such as Cx3cr1 and Ly86.

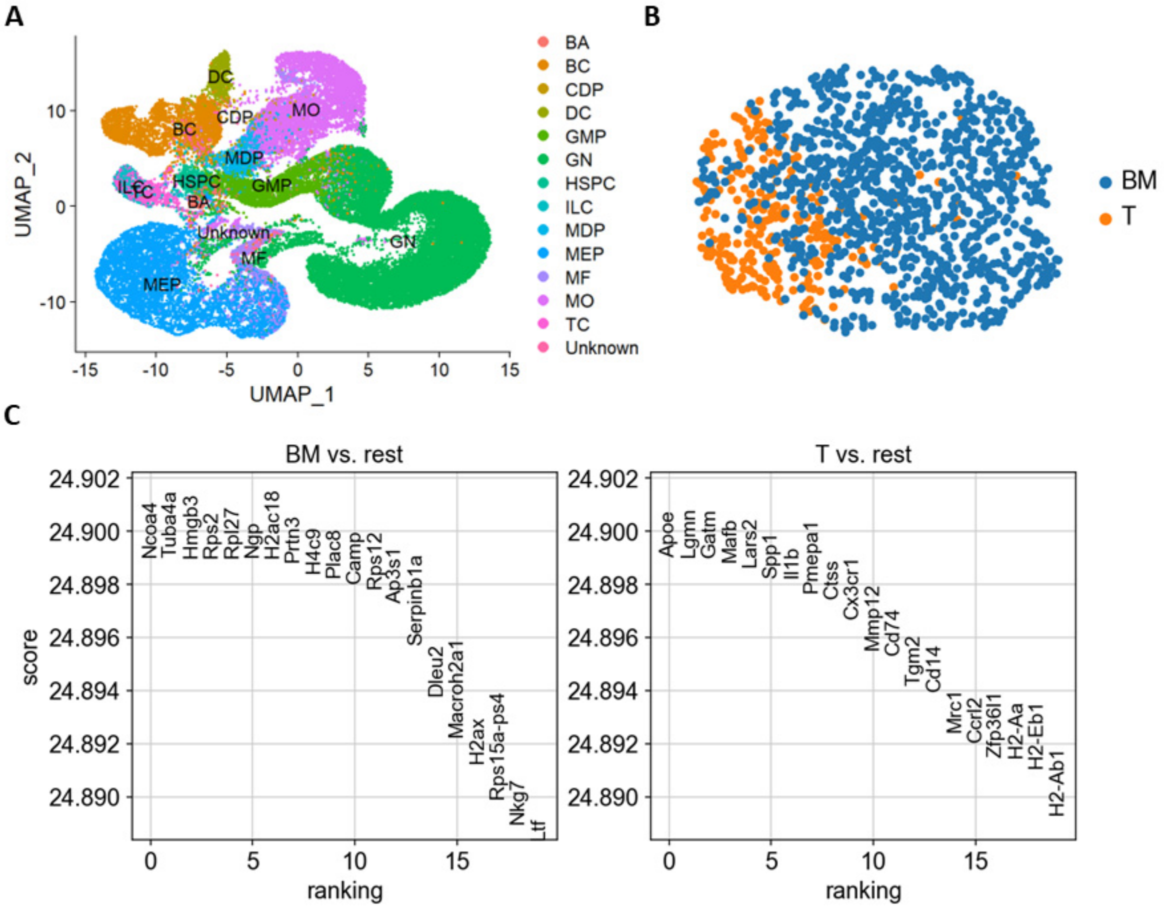

Figure S8: The comparison of MDPs between 4T1 tumors and normal bone marrow, related to Figure 2. (A) Different cells from the bone marrow were characterized by ImmGen, identifying 984 MDPs. (B) the integration between bone marrow and tumors using the scVI model. (C) The ranking genes ineach cluster relative to the other using the Wilcoxon rank-sum test. The marker genes of the bone marrow cluster (left) are associated with differentiation, while the cells found in tumors (right) largely express M2-like monocytic genes. BM, bone marrow; T, tumor.

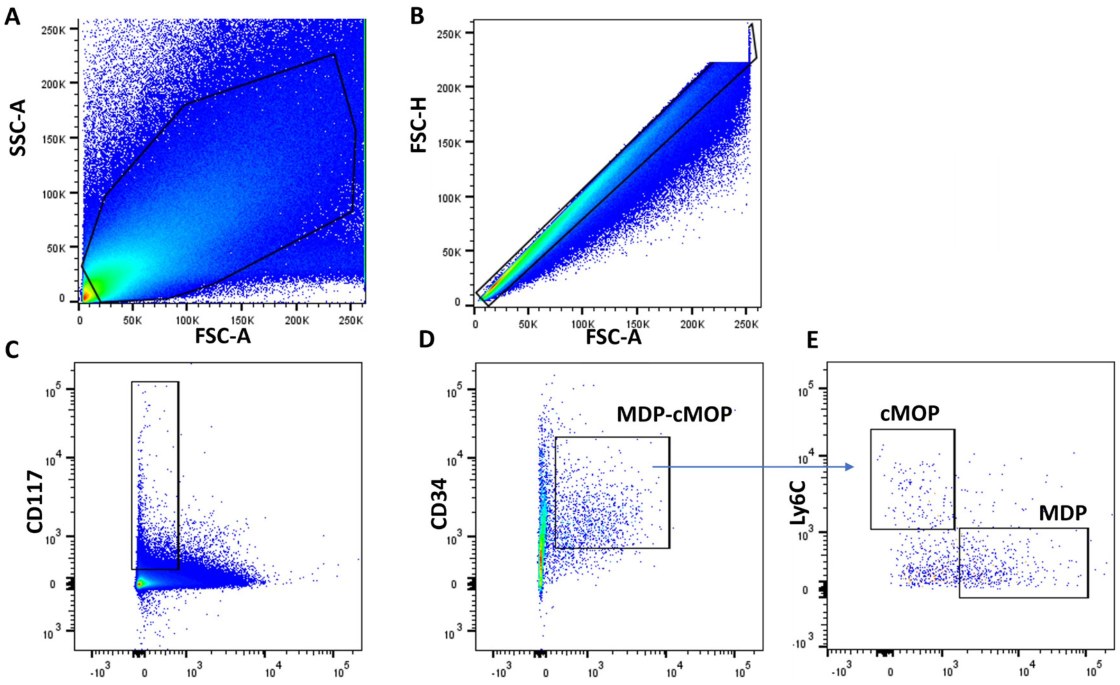

Figure S9: MDP-cMOPs flow cytometry gating strategy, related to Figure 2. (A-E) Representative flow cytometry plots taken from tumor single cell suspensions of BALB/c mice bearing 4T1m breast carcinoma tumors. The analysis was performed when first gating on SSC and FSC, collecting the majority of events in the samples (A), while eliminating cell doublets (B). MDP-cMOPs were defined with a gate positive to CD117 and negative to Sca-1 (C) and subsequently positive to CD34 and CD115 (D), together representing the MDP-cMOP population. CD135 and Ly6C are also shown to distinguish MDP from cMOP (E), but they were excluded from the analysis due to the rare events.

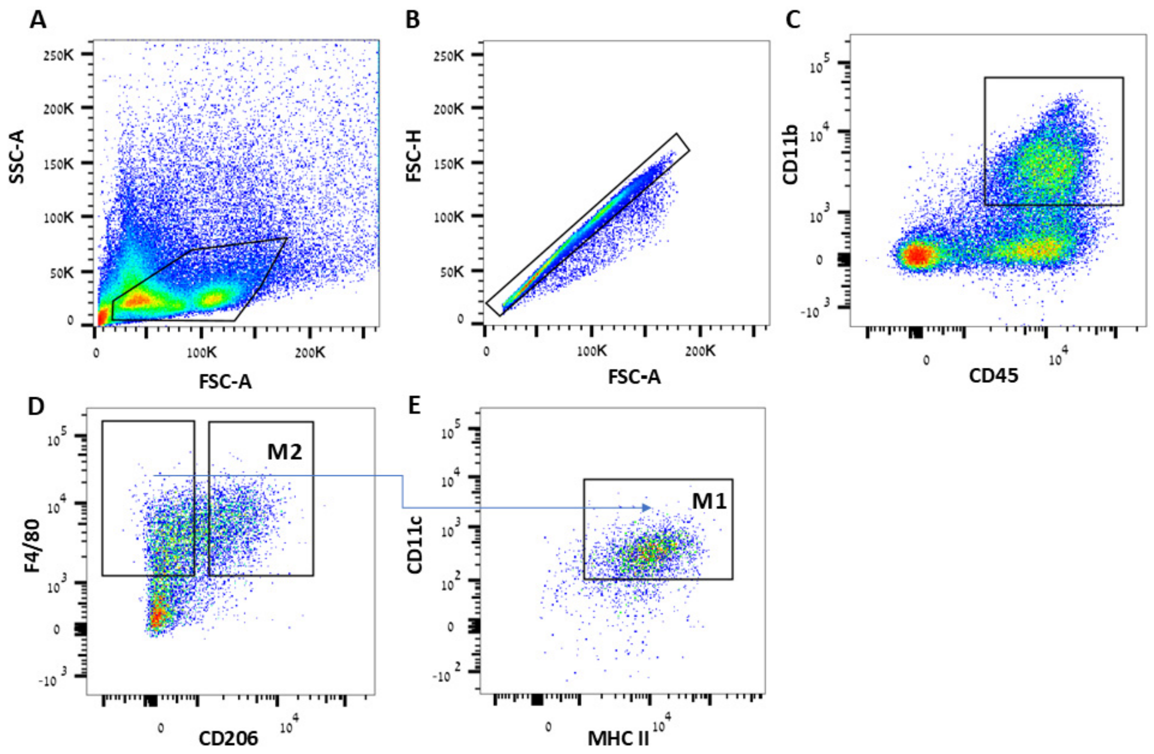

Figure S10: Macrophage flow cytometry gating strategy, related to Figure 2. (A-E) Representative flow cytometry plots taken from tumor single cell suspensions of BALB/c mice bearing 4T1m breast carcinoma tumors. The analysis was performed when first gating on SSC and FSC, collecting the majority of events in the samples (A), followed by the elimination of doublets (B). From these events, CD45+/CD11b+ cells were gated (C). Macrophages were then identified by gating on M2-like macrophages (CD206+/F4/80+) and M1-like macrophages, characterized by CD206-/F4/80+ (D), followed by MHCII+/CD11c+ markers (E).

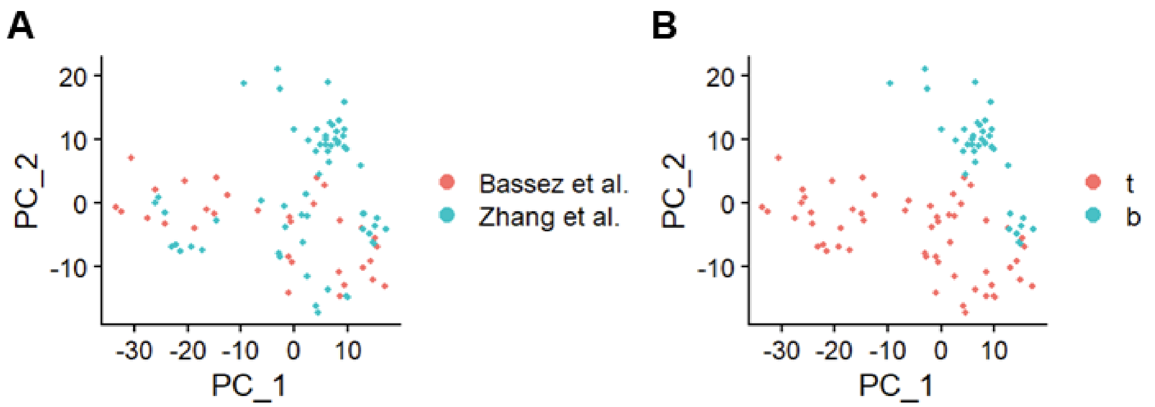

Figure S11: Assessment of batch effects in human scRNA-seq analysis, related to Figure 4. (A-B) A PCA plot of the mergedtriple negative breast cancer, human data. Points represent individual cells, labeled by their source dataset (A). Tumor-derived cells (denoted “t”) from the two datasets merge smoothly, while blood cells from the Zhang et al. data set (denoted “b”) are distinct from these cells (B).

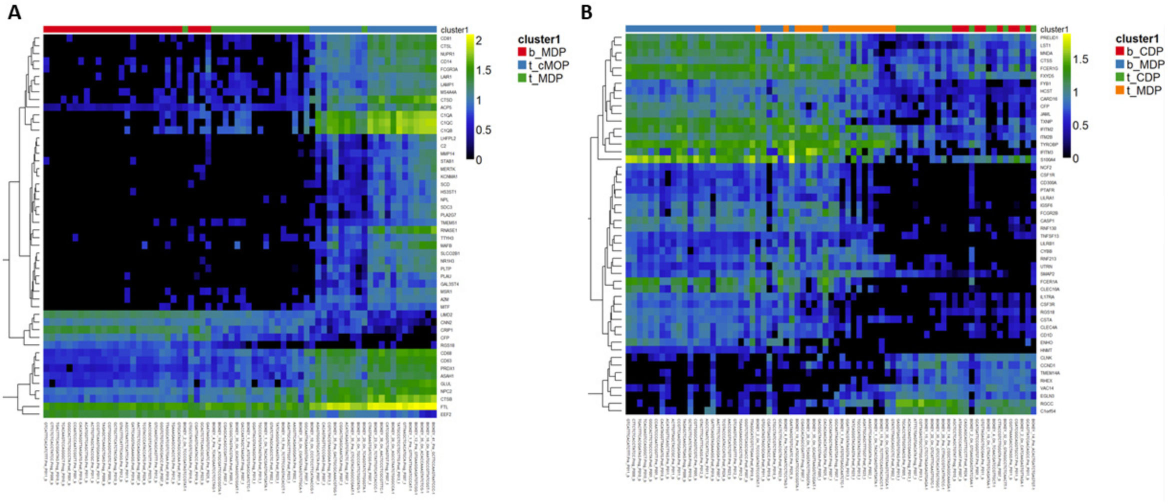

Figure S12: Heatmap of the 50 top genes with varied expression over pseudotime, related to Figure 4. (A-B) Gene changes along the trajectory from MDPs to cMOP (A) or to CDPs (B) are shown.

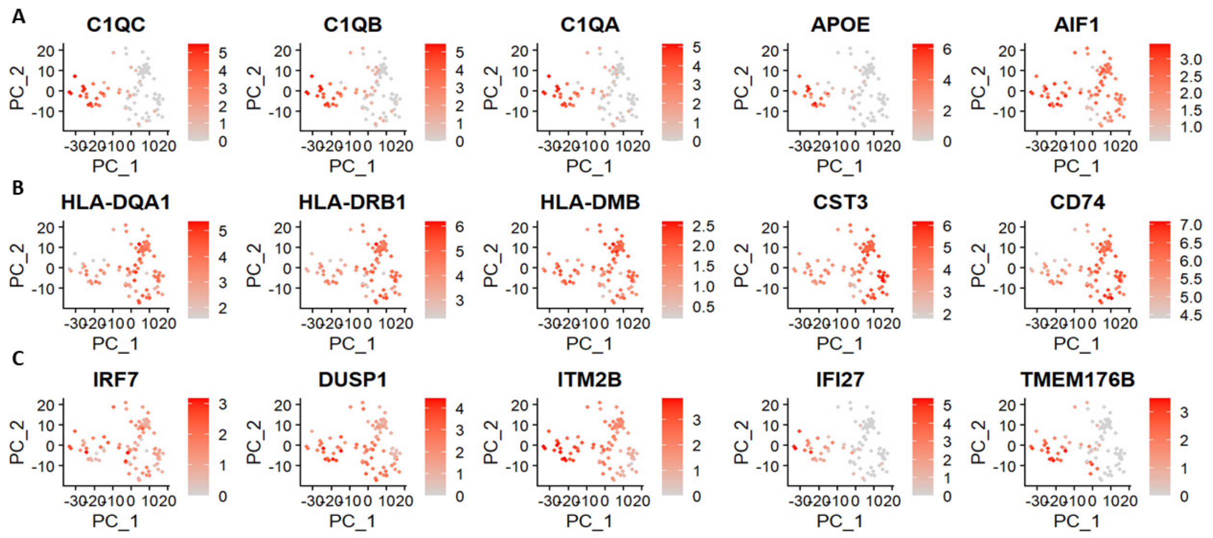

Figure S13: Gene signatures of MDP-cMOP correlate with the immunosuppressive fate of MDPs, based on murine analysis, related to Figure 4. (A-B) Binned, normalized expression of genes in cells, asshown in Figure 3A-C, highlighting pathways involved in the differentiation of MDPs to cMOPs (A) and CDPs (B). (C) Binned, normalized genes associated with MDPs and cMOPs in humans, compared to those in mice, are shown.

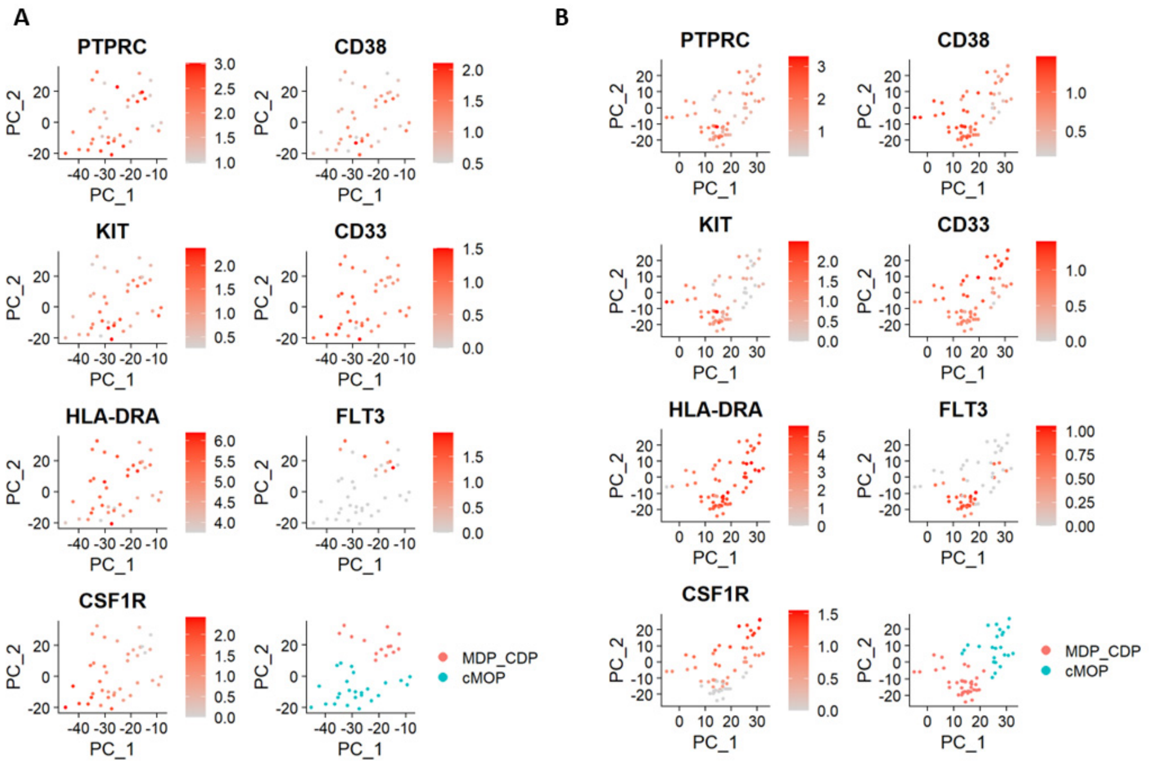

Figure S14: MDPs are found in the tumors of colorectal and lung cancer patients, related to Figure 4. (A-B) Binned,normalized expression of genes from cells representing CDP, MDP and cMOP in colorectal cancer (A) and lung cancer (B) patients. PCA plots colored by the surface markers of MDPs and their committed subsets are shown.

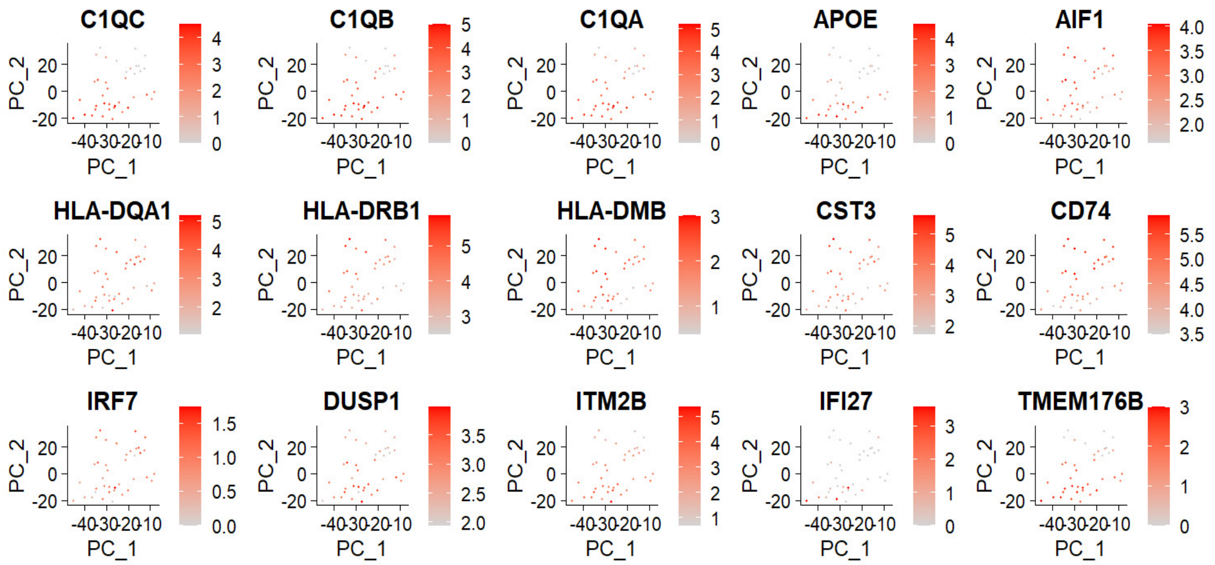

Figure S15: Gene signatures of MDP-cMOP correlate with the immunosuppressive fate of MDPs, based on murine analysis (Colorectal cancer), related to Figure 4. (A-B) Binned, normalized genes associated with MDPs and cMOPs in humans, compared to those in mice, are shown.

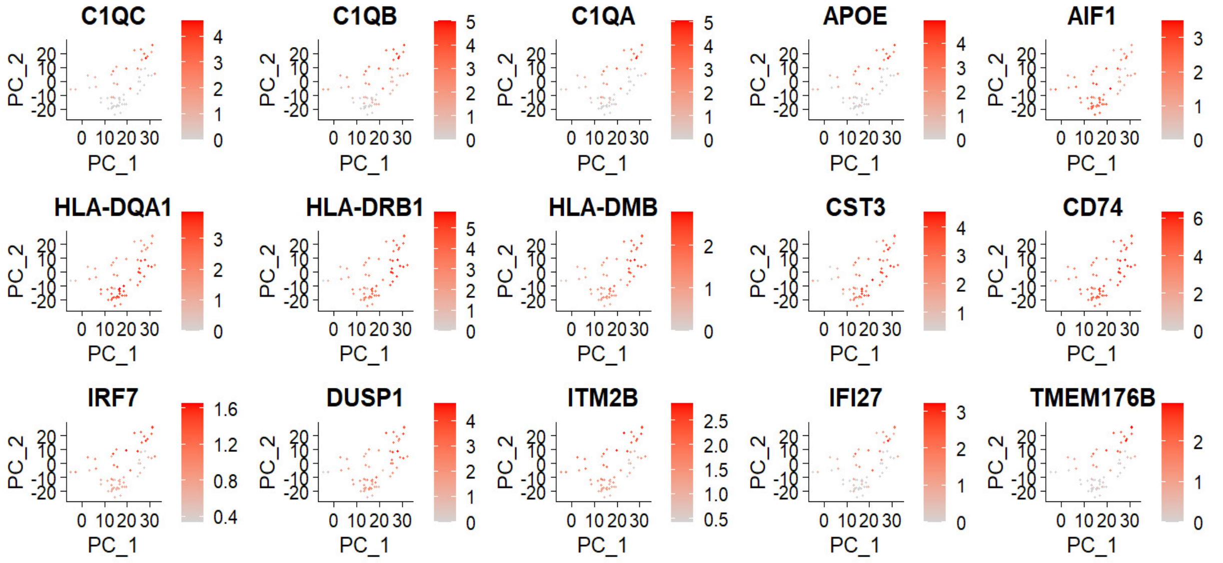

Figure S16: Gene signatures of MDP-cMOP correlate with the immunosuppressive fate of MDPs, based on murine analysis (Lung cancer), related to Figure 4. (A-B) Binned, normalized genes associated with MDPs and cMOPs in humans, compared to those in mice, are shown.

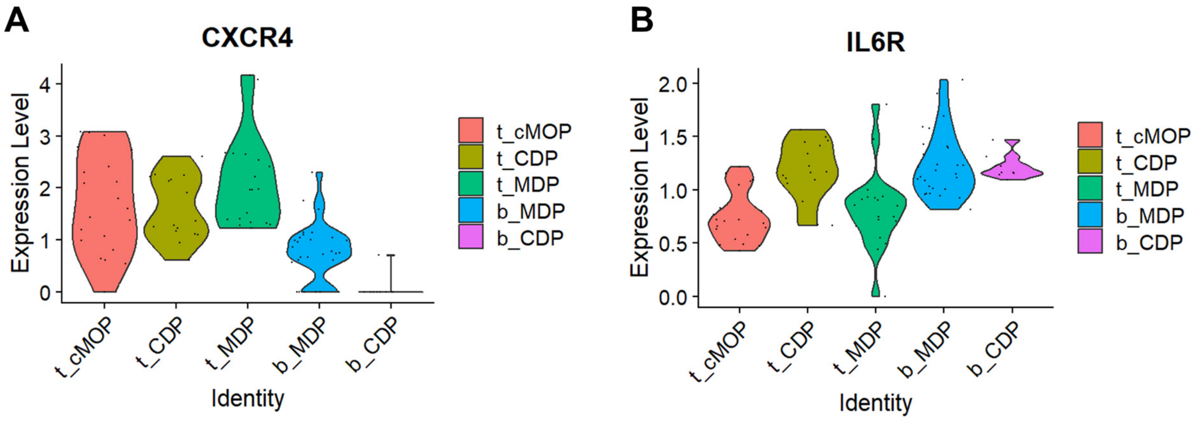

Figure S17: CXCR4 and IL6R expression in MDP, CDP and cMOP obtained from tumors and blood of breast cancer patients, related to Figure 5. The Violin plots showing the expression of CXCR4 (A) and IL6R (B) in human cells across the indicated populations (as shown in the figure). t, tumor; b, blood.

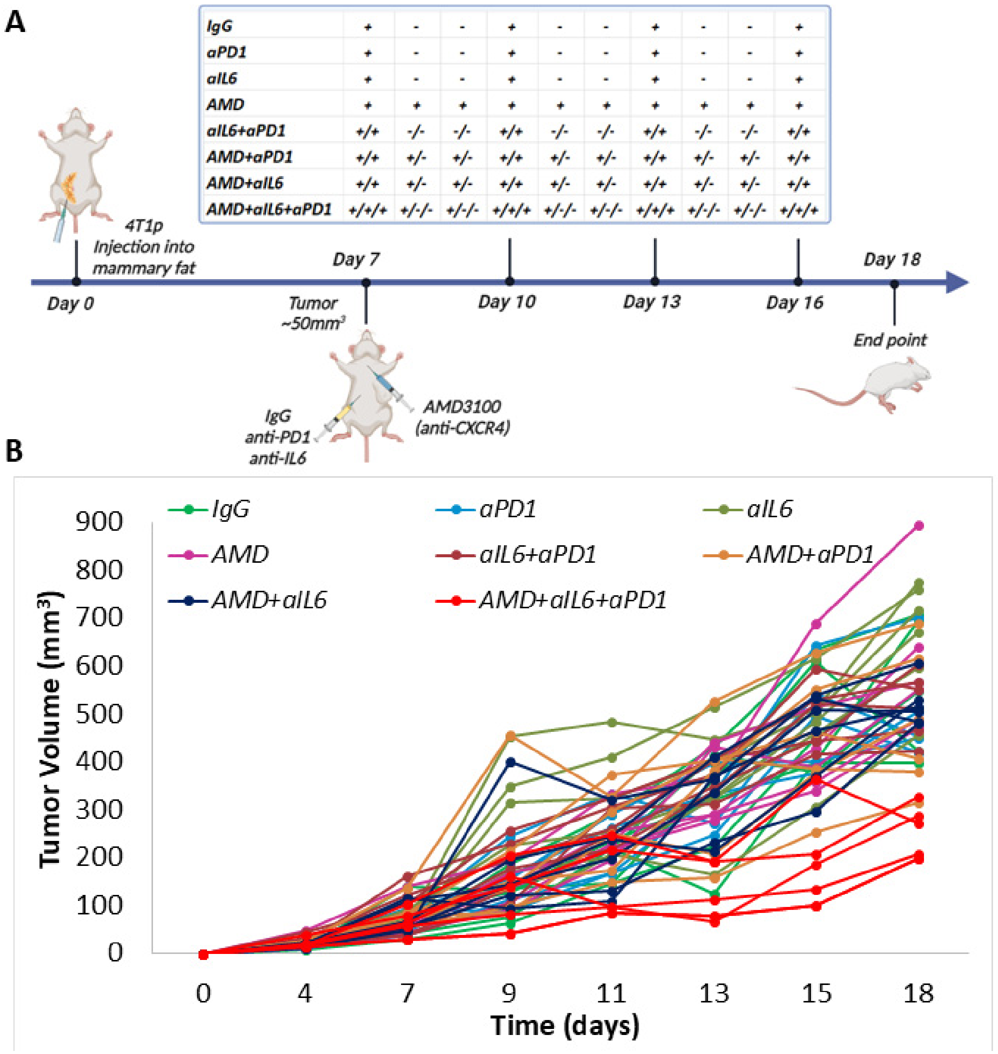

Figure S18: Spider plots of tumor growth following treatment with anti-PD1, anti-IL6, and AMD3100, or their combination thereof, related to Figure 5. (A) A schematic overview of the experimental set-up for 4T1p tumors treated with anti-PD1 (αPD1), anti-IL6 (αIL6), AMD3100 (AMD), or their combination. (B) Raw, individual tumor growth profiles of 4T1p breast tumor groups, colored by treatment are shown as a spider plot.

Supplemental Tables

Table S5

Cell Type	Phenotype	
	**Human**	**Mouse**
**MDP**	CD45RA$^+$ CD38$^+$ CD117$^+$ CD33$^+$ CD115$^+$ CD135$^+$HLA-DR$^+$	SCA-1$^-$ CD117$^+$ CD34$^+$ CD115^{hi} CD135$^+$
**CDP**	CD45RA$^+$ CD38$^+$ CD117$^+$ CD33$^+$ CD115$^-$ CD135$^+$HLA-DR$^+$	SCA-1$^-$ CD117$^+$ CD34$^+$ CD115$^-$ CD135$^+$
**cMOP**	CD45RA$^+$ CD38$^+$ CD117$^+$ CD33$^+$ CD115$^+$ CD135$^-$	SCA-1$^-$ CD117$^+$ CD34$^+$ CD115^{hi} CD135$^-$
**M-MDSC**	CD33$^+$ CD11B$^+$ CD14$^+$ CD15- CD66b$^+$ HLA-DR$^-$	GR-1$^+$ CD11B$^+$ LY6C^{hi} LY6G$^-$
**G-MDSC**	CD33$^+$ CD11B$^+$ CD14$^-$ CD15$^+$ CD66b$^+$ HLA-DR$^-$	GR-1$^+$ CD11B$^+$ LY6C^{lo} LY6G$^+$
**M1**	CD68$^+$ CD80$^+$ CD86$^+$ CD206$^-$ CD163$^-$	CD45$^+$ CD11B$^+$ F4/80$^+$ CD206$^+$ CD11C$^+$MHCII$^+$
**M2**	CD68$^+$ CD80$^-$ CD86$^-$ CD206$^+$ CD163$^+$	CD45$^+$ CD11B$^+$ F4/80$^+$ CD206$^+$ CD11C$^-$MHCII$^-$

Table S5: Surface markers define different immune cell types in human and mouse, related to Figure 2. Surface markers expressed by different immune cell types are listed in the table for both human and mouse. Underlined surface markers were specifically analyzed in murine cells using flow cytometry or scRNA-seq of cancer patients. MDP, monocytic dendritic progenitor; CDP, common dendritic progenitor; cMOP, common monocytic progenitor; M-MDSC, monocytic-myeloid derived suppressor cells; G-MDSC, granulocytic- myeloid derived suppressor cells; M1, proinflammatory macrophages; M2, immunosuppressive macrophages.
